# Non-invasive imaging of Young’s modulus and Poisson’s ratio in cancers *in vivo*

**DOI:** 10.1038/s41598-020-64162-6

**Published:** 2020-04-29

**Authors:** Md. Tauhidul Islam, Songyuan Tang, Chiara Liverani, Sajib Saha, Ennio Tasciotti, Raffaella Righetti

**Affiliations:** 1 Department of Radiation Oncology, Stanford University, Stanford, CA 94305 USA; 2 Department of Electrical and Computer Engineering, Texas A&M University, College Station, Texas 77840 USA; 3 Osteoncology and Rare Tumors Center, Istituto Scientifico Romagnolo per lo Studio e la Cura dei Tumori (IRST) IRCCS, Meldola, Italy; 4 Department of Civil Engineering, Texas A&M University, College Station, Texas 77840 USA; 5 Center of Biomimetic Medicine, Houston Methodist Research Institute, 6670 Bertner Avenue, Houston, TX 77030 USA

**Keywords:** Diagnosis, Ultrasonography

## Abstract

Alterations of Young’s modulus (YM) and Poisson’s ratio (PR) in biological tissues are often early indicators of the onset of pathological conditions. Knowledge of these parameters has been proven to be of great clinical significance for the diagnosis, prognosis and treatment of cancers. Currently, however, there are no non-invasive modalities that can be used to image and quantify these parameters *in vivo* without assuming incompressibility of the tissue, an assumption that is rarely justified in human tissues. In this paper, we developed a new method to simultaneously reconstruct YM and PR of a tumor and of its surrounding tissues based on the assumptions of axisymmetry and ellipsoidal-shape inclusion. This new, non-invasive method allows the generation of high spatial resolution YM and PR maps from axial and lateral strain data obtained via ultrasound elastography. The method was validated using finite element (FE) simulations and controlled experiments performed on phantoms with known mechanical properties. The clinical feasibility of the developed method was demonstrated in an orthotopic mouse model of breast cancer. Our results demonstrate that the proposed technique can estimate the YM and PR of spherical inclusions with accuracy higher than 99% and with accuracy higher than 90% in inclusions of different geometries and under various clinically relevant boundary conditions.

## Introduction

Pathological changes typically alter the mechanical behavior of tissues. To describe the mechanical behavior of linear elastic tissues, at least two parameters are required - namely the Young’s modulus (YM) and the Poisson’s ratio (PR). YM is a mechanical parameter than can be used as a measure of tissue stiffness while PR provides a measure of tissue compressibility (i.e., the relative volume change of a material to a pressure change).

Currently, a few imaging modalities are capable of generating YM maps of tissues *in vivo* and non-invasively while there are no available methods to non-invasively image the PR in tissues. Ultrasound elastography (USE)^1^, ultrasound shear wave elastography (SWE)^2^ and magnetic resonance elastography (MRE)^3^ techniques have shown to be capable of generating YM images^4–6^. Recent studies have demonstrated the feasibility of imaging the lateral-to-axial strain ratio, also referred to as “effective PR” (EPR), in tissues using elastography^7,8^, but not the actual, underlying PR of the tissue.

In most of the medical elasticity imaging techniques retrievable in the literature, the YM of the tissue is reconstructed using two fundamental assumptions: (1) that the tissue (tumor and surrounding tissue) behaves as a perfectly linearly elastic solid, and (2) that the tissue is incompressible or nearly incompressible^4–6^. The first assumption allows these methods to estimate the YM of the tissue from knowledge of the instantaneous strain in response to the applied compression. The PR of the tissue, which is also needed to correctly estimate the YM of the tissue, is not estimated. Based on the second assumption, PR is assumed to be a given value, typically 0.495/0.499995/0.45^4–6^. In regard to the first assumption, it is now widely believed that tissues can be more realistically represented using poroelastic models as opposed to linearly elastic models^9–11^. Thus, their strain response under loading varies with time. In that case, YM and PR should be estimated from the strain response at steady state, when the material is fully relaxed^12^, rather than from the instantaneous response. In fact, in soft tissues, the YM estimated from the instantaneous strain can be significantly higher (2–4 times) than the true YM value as shown in Bayat *et al*.^13^. In regard to the second assumption, it has been demonstrated by a number of prominent research labs that tissues (including tumors) exhibit varying degree of compressibility. In the works of Stylianopoulos *et al*.^9^, Mpekris *et al*.^14^ and Fung^15^, the PR of normal tissue was assumed 0.2. PR of cancers was assumed 0.2 (compressible)/0.45 (incompressible) in works of Stylianopoulos *et al*.^9^, Roose *et al*.^16^ and Netti *et al*.^17^. Recently, Nia *et al*.^12^ assumed a PR value for soft tissue and tumor of 0.1 to compute the residual stress inside the tumor. In other works^18,19^, the authors reported values of PR for the soft tissue ranging between 0.3 and 0.45. Given the broad range of PR values in soft tissue and tumors reported in the literature, the assumption that PR is constant and equal to 0.5 or a value close to 0.5 is, in many cases, unrealistic. Accurate determination of PR is crucial to obtain accurate estimates of YM and also to correctly reconstruct other mechanical parameters. For example, accurate knowledge of YM and PR is essential for the quantification of transport parameters such as vascular permeability and interstitial permeability, which are known to be of great clinical value^10^. Finally, PR may change with the onset of different diseases^8^, and this information could prove useful clinically.

In addition to the tissue incompressibility assumption, most YM reconstruction methods retrievable in the literature present a number of other limitations. Estimation of the mechanical properties of tumors is inherently a three-dimensional problem. While a few three-dimensional YM reconstruction methods have been reported in the literature^6,20–22^, in most of previously reported YM reconstruction studies, the models are two-dimensional and operate under the assumption of plane strain/plane stress conditions^4,23^. Most of retrievable methods assume specific boundary conditions such as total uniformity of the background, stress-free lateral boundaries etc., which are often not applicable to tissues. Most of these methods perform well for inclusions of specific shape such as disk (2D)/sphere (3D)^4,20,23^ but their performance deteriorates for inclusions of other shape such as ellipse. As the available YM reconstruction methods rely only on the axial strain contrast, they cannot perform efficiently when the inclusion/background YM contrast is larger than 10 or when the inclusion is softer than the background^6,20,23^. In many cases, the inclusion is assumed to be very small so that ratios such as the sample radius to inclusion radius ratio, the compressor radius to inclusion radius ratio and/or the distance between applied force and inclusion to inclusion radius ratio are greater than a predefined value^20^. Determination of heterogeneous distribution of YM inside the inclusion and background is another challenge^24^. Most of these methods fail to reconstruct the YM accurately in case of non uniform axial compression, which occurs frequently in clinical elastography experiments.

Poroelastography is an elastographic technique that aims at assessing the poroelastic behavior of tissues by analyzing the temporal and spatial distributions of the local strains (and related parameters) resulting in the tissue under compression^8,25^. In an ultrasound poroelastography experiment, the tissue is compressed for a certain time interval while a series of radio frequency (RF) data is acquired. From the RF data, time-dependent axial and lateral strain elastograms are computed. Since, at steady state, the tissue behaves as a linear elastic material, from knowledge of the steady state axial and lateral strain distributions, it is possible to determine the local YM and PR of the poroelastic tissue using formulations based on linear elasticity theory.

In this paper, we present a poroelastography method that allows reconstruction of both YM and PR based on Eshelby’s inclusion theoretical formulation^26,27^. Our proposed method overcomes the aforementioned limitations of previously proposed YM reconstruction methods. It allows simultaneous quantification and imaging of the YM and PR in both the inclusion and the background based on the assumptions of axisymmetry and ellipsoidal shape, is robust to variations in inclusion shape and works for a wide range of inclusion/background YM contrasts (0.1–50) and under a wide range of complex boundary conditions. The proposed method is then used to image and quantify YM and PR distributions in cancers *in vivo*.

## Results

### Simulations

The YM distributions in simulated samples with different mechanical properties reconstructed using the proposed method were compared with results obtained using two previously proposed 3D reconstruction methods, which here are referred to as “3DB”^20^ and “3DS”^6^. Eight samples with inclusions having different shape (Z1–Z8), nine samples with different inclusion/background YM contrasts (fixed inclusion/background PR contrast) (X1–X9), three samples with different boundary conditions (B1–B3), three samples with inclusions having different YM heterogeneity percentages (H1–H3), four samples with different non-uniform loadings (R1–R4) and thirteen samples with different inclusion/background YM and PR contrasts (A–M) were simulated and analyzed. Selected results are shown in Figs. S3 and S4. Fig. S3 shows YM images of the simulated samples A–D reconstructed using the three reconstruction methods. The corresponding PR images created using our proposed method are shown in Fig. S4.

**Figure 1:**
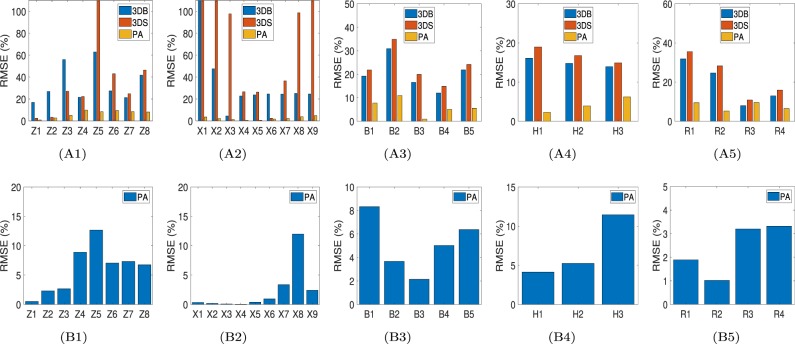
Analysis of error in estimation of YM and PR when using different methods and under different simulation conditions. PA stands for: proposed approach. (**A1** and **B1**) RMSEs in estimating the YM and PR of inclusions of different shapes using different methods (YM inclusion/background contrast (CTYM) is 3). Z1–Z8 samples have spherical, prolate, oblate, cylindrical, penny, tetragon, pentagon and hexagon inclusions, respectively. (**A2** and **B2**) RMSEs in estimating the YM and PR of spherically shaped inclusions for different CTYM values. X1–X9 samples have CTYM of 0.1, 0.2, 0.5, 3, 5, 15, 25, 50 and 100, respectively. (**A3** and **B3**) RMSEs in estimating the YM and PR of spherically shaped inclusions with a CTYM of 3 for different complex boundary conditions. B1 has zig-zag stiff background, B2 has 14 different shaped inclusions in background, B3 has strip of hard tissue on top of the tumor and B4 and B5 have multiple layers if tissue on top of the tumor. (**A4** and **B4**) RMSEs in estimating the YM and PR of the spherically shaped inclusions with different heterogeneity conditions and CTYM of 3. H1–H3 samples have inclusions of 10, 20 and 30% heterogeneity, respectively. (**A5** and **B5**) RMSEs in estimating the YM and PR of spherically shaped inclusions with CTYM of 3 under non-uniform loading. R1 and R2 have reduction of load by 10 and 20%, respectively from the center to the periphery of the compressor plate. In case of R3 and R4, the load was increased by 10 and 20%, respectively.

The percent root mean squared error (RMSE) occurring when reconstructing the YM distribution in inclusions of different shape using the 3 approaches are shown in Fig. 1(A1). We observe that, within the results obtained using the proposed approach, the highest RMSE is observed when the shape of the inclusion is cylindrical (9.91%) and the lowest (0.8%) when the shape of the inclusion is spherical. In comparison, the RMSE associated to the other two YM reconstruction methods are much higher than the one associated to the proposed method and typically higher than 20% for inclusions of all shapes. The RMSEs occurring when reconstructing the PRs using the proposed approach are shown in Fig. 1(B1). These are found to be less than 9% in most of the simulated samples.

The RMSEs computed for the three methods in the case of inclusions having different YM contrast with respect to the background are reported in Fig. 1(A2). We see that the RMSE associated to the proposed approach is below 5% for contrast in the range 0.1–100, whereas the RMSEs for the 3DB and 3DS approaches are higher than 20% in most cases.

A typical problem of elastography-based reconstruction methods is the effect of boundary conditions on the reconstructed mechanical parameters. The RMSEs computed when the YM of the inclusion is reconstructed using data obtained with different boundary conditions are shown in Fig. 1(A3). We see that, even in the case of very complex boundary conditions, the proposed approach can reconstruct the YM with about 90% accuracy. The other two reconstruction methods show higher RMSEs when compared to the proposed one.

Effect of heterogeneity in the YM distribution inside the inclusion on the reconstructed parameters has been investigated, and the results are reported in Fig. 1(A4). Sample H3 has the highest heterogeneity - YM reduces by 30% from the center to the periphery of the inclusion. The proposed method is capable of reconstructing the YM of the inclusion with high accuracy (>94%) in all cases analyzed in this study whereas the other two approaches introduce error above 14% in most cases (see Figs. S8 and S9 for reconstructed YM and PR maps by different methods).

The results related to the non-uniform compression conditions are shown in Fig. 1(A5). Once again, the proposed method is robust to load variations, as opposed to the other two methods.

**Figure 2:**
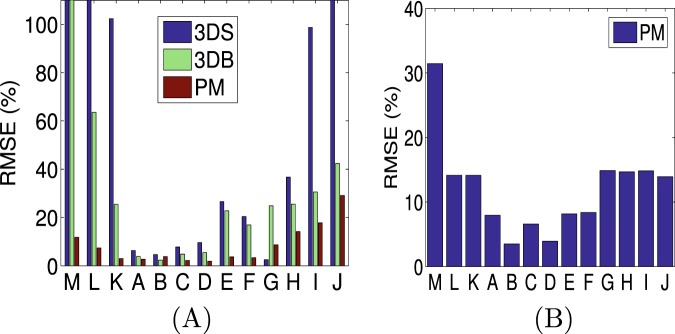
(**A**) Root mean squared errors (RMSE) of estimated YM images and (**B**) RMSEs of estimated PR images for samples A-M using ultrasound simulated data. RMSEs greater than 100% have been masked to 100%. Samples A-J have inclusion harder than the surrounding background, and K-M have inclusions softer than the surrounding background. RMSE is higher in case of samples with soft inclusions for all the methods.

In Fig. 2(A), we report the RMSEs of the estimated YM images using the three reconstruction techniques for thirteen samples A-M when using simulated ultrasound data, and the RMSEs of the estimated PRs in the same samples using the proposed technique are shown in Fig. 2(B). The reconstructed YM images of sample A–D are shown in Fig. S13 and the reconstructed PRs of samples A–D are shown in Fig. S14. We see from Fig. 2 that the error incurred in all the reconstruction methods increases as the inclusion/background YM contrast increases. However, the RMSE for the proposed method is below or around 15% for inclusion/background YM contrasts up to 50 (sample H). The RMSE for the estimated PR also increases as the inclusion/background YM contrast increases but remains around 10% even in case of a contrast of 100 (sample J). The other two methods can introduce errors greater than 25% even in case of a contrast as low as 3 (sample E). The RMSE for all methods increases for the samples with a soft inclusion (samples K-M). However, the error is significantly lower for the proposed method in comparison to the other two techniques. For sample M, where the inclusion is 10 times softer than the background, the RMSEs are higher than 100% for 3DB and 3DS methods, while the RMSE for the proposed technique is below 10%. These results prove that the proposed approach is more accurate, more precise and more robust than previously proposed 3D YM reconstruction methods and has the advantage to provide estimates of both the YM and the PR distributions.

### Controlled experiments and mechanical testing

**Figure 3:**
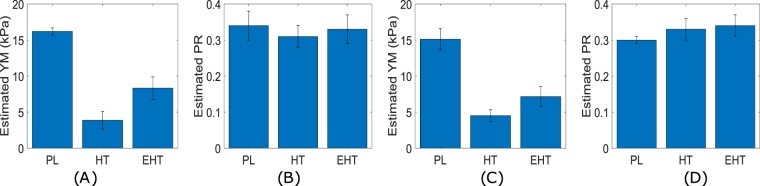
Estimated YM (**A**) and PR (**B**) of the polyacrylamide (PL), hard tofu (HT) and extra hard tofu (EHT) samples from mechanical testing. Estimated YM (**C**) and PR (**D**) of PL, HT and EHT from elastography experiments.

The mean and standard deviation of YM and PR measurements obtained from 60 mechanical tests (10 tests/sample) performed on two hard and two extra hard tofu samples and two polyacrylamide samples are shown in Fig. 3(A,B). We see that polyacrylamide has the highest YM mean among the three materials. The extra hard tofu has almost twice the YM mean of hard tofu. The mean PR of the hard tofu and the mean PR of the extra hard tofu are close in values and slightly higher than the PR mean of the polyacrylamide.

**Figure 4:**
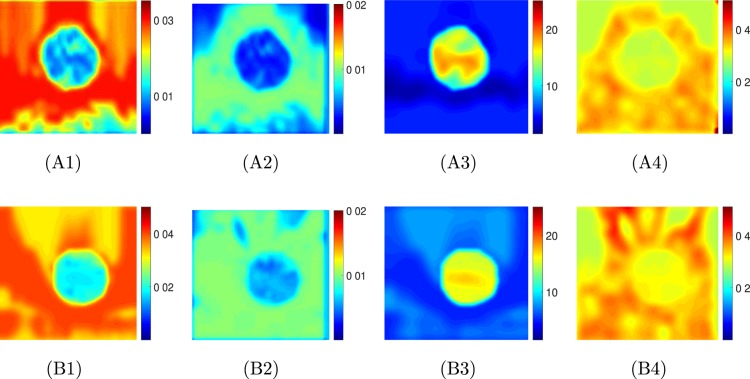
Estimated axial strain (1), lateral strain (2), YM (in kPa) map (3) and PR map (4) from controlled elastography experiment performed on a cylindrical sample made of (**A**) hard tofu (**B**) extra hard tofu with a polyacrylamide inclusion. The applied compressions were 0.152 kPa and 0.253 kPa, respectively.

Figure 4 shows selected results from elastography experiments performed on the tofu samples with cylindrical inclusion made of polyacrylamide. In Fig. 4(A1–A4,B1–B4), the estimated axial strain, lateral strain, reconstructed YM and PR distributions are shown for two samples with background made of hard tofu and extra hard tofu, respectively (the inclusion is made of polyacrylamide in both cases). From this figures, we see that, overall, YM maps present uniform values both inside and outside the inclusion. The PR maps are noisier than the YM maps, and the PR varies in the background in the range 0.3–0.4. The mean and standard deviation of the reconstructed YM and PR distributions of hard tofu and extra hard tofu and polyacrylamide as obtained from all eight controlled elastography experiments can be found in Fig. 3(C,D). By comparing these values with that obtained from mechanical testing (Fig. 3(A,B)), we see that our reconstructed YM and PR have error lower than 15% across all the materials, despite the technical challenges associated with both the mechanical tests and the elastography experiments. In the supplementary material (section 20), we show also results obtained from a commercially available breast phantom containing nearly incompressible inclusions, i.e., PR 0.5 (Fig. S28). The proposed method can accurately estimate both the YM and PR also in the case of incompressible inclusions.

### *In vivo* experiments

**Figure 5:**
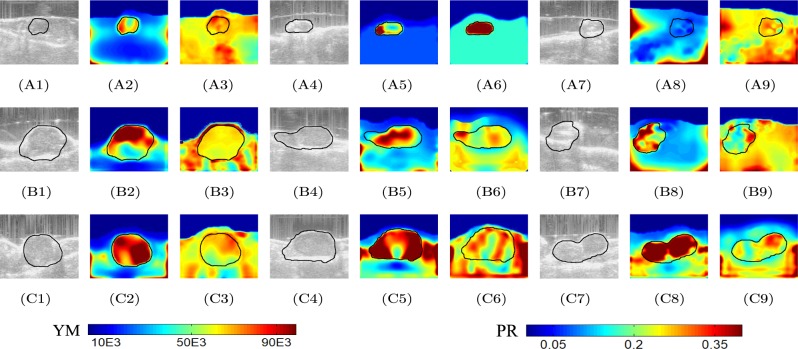
B-mode images of untreated mouse #1 at three time points (week 1, week 2, week 3) are shown in (**A1**), (**B1**) and (**C1**). Reconstructed YM (in Pa) and PR distributions at the same time points are shown in (**A2**), (**B2**) and (**C2**), and (**A3**), (**B3**) and (**C3**), respectively. B-mode images of untreated mouse #2 at three time points (week 1, week 2, week 3) are shown in (**A4**), (**B4**) and (**C4**). Reconstructed YM and PR distributions at the same time points are shown in (**A5**), (**B5**) and (**C5**), and (**A6**), (**B6**) and (**C6**), respectively. B-mode images of untreated mouse #3 at three time points (week 1, week 2, week 3) are shown in (**A7**), (**B7**) and (**C7**). YM and PR distributions at the same time points are shown in (**A8**), (**B8**) and (**C8**), and (**A9**), (**B9**) and (**C9**), respectively. In all cases, YM increases from week 1 to week 3, indicating increased stiffness of the cancers as they grow. The PRs do not change significantly with time (≈0.3).

B-mode images and reconstructed YM and PR distributions obtained from data acquired from three untreated mice at three different time points (week 1, week 2 and week 3) are shown in Fig. 5(A1–A9, B1–B9 and C1–C9). We see from this figure that, in general, the YM increases significantly from week 1 (A2, A5, A8) to week 3 (C2, C5, C8) in the untreated mice, while the PR values do not appear to significantly change with time.

**Figure 6:**
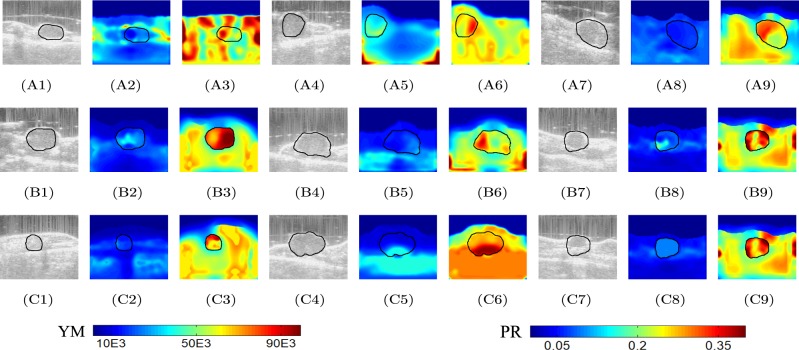
B-mode images of treated mouse #1 at three time points (week 1, week 2, week 3) are shown in (**A1**), (**B1**) and (**C1**). Reconstructed YM (in Pa) and PR distributions at the same time points are shown in (**A2**), (**B2**) and (**C2**), and (**A3**), (**B3**) and (**C3**), respectively. B-mode images of treated mouse #2 at three time points (week 1, week 2, week 3) are shown in (**A4**), (**B4**) and (**C4**). Reconstructed YM and PR distributions at the same time points are shown in (**A5**), (**B5**) and (**C5**), and (**A6**), (**B6**) and (**C6**), respectively. B-mode images of treated mouse #3 at three time points (week 1, week 2, week 3) are shown in (**A7**), (**B7**) and (**C7**). Reconstructed YM and PR distributions at the same time points are shown in (**A8**), (**B8**) and (**C8**), and (**A9**), (**B9**) and (**C9**), respectively. Overall, the YM values of the treated mice are significantly lower than that of the untreated mice, whereas the PR values of the treated mice are higher than that of the untreated ones. The reduction/non-increment of stiffness of the treated tumors may be a sign of the efficacy of the treatment in controlling the growth of the cancer.

B-mode images and reconstructed YM and PR distributions obtained from data acquired from three treated mice at three different time points (week 1, week 2 and week 3) are shown in Fig. 6(A1–A9, B1–B9 and C1–C9). We see from this figure that, in most treated mice, the YM decreases or remains the same in time. Also, the YM contrast between cancer and background tissue is not as high as in the case of the untreated mice. The PR values are in the range 0.3–0.4 in most of the cases.

**Figure 7:**
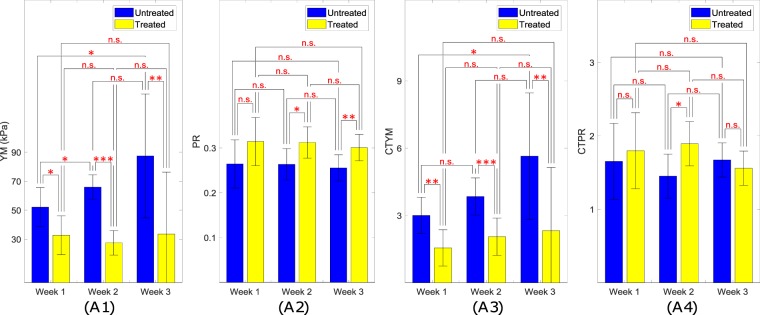
(**A1**) Mean YM values for the treated and untreated mice at week 1, week 2 and week 3. (**A2**) Mean PR values for the treated and untreated mice at week 1, week 2 and week 3. (**A3**) Mean YM contrast between tumor and normal tissue for treated and untreated mice at week 1, week 2 and week 3. (**A4**) Mean PR contrast between tumor and normal tissue for treated and untreated mice at week 1, week 2 and week 3. “n.s.” stands for “not statistically significant”; one, two and three stars correspond to *p*-value less than 0.05, 0.01, 0.001, respectively. The mean values of YM and CTYM of the tumors increase from week 1 to week 3 for untreated mice and remain almost constant for the treated ones. Mean values of PR and CTPR of the tumors are consistently higher for the treated tumors than the untreated ones.

The mean values with the corresponding standard deviations of YM for twelve treated mice and seven untreated mice used in our *in vivo* experiments at the three different time points (week 1, week 2 and week 3) are shown in Fig. 7(A1). In the first week, the mean YM of the tumors in the untreated mice was found to be below 50 kPa. In the second week, the mean YM of the untreated tumors increased significantly (above 60 kPa) and in the third week was found to be above 75 kPa. The mean YM of the tumors in the treated mice at the three different weeks was found to be close to 25 kPa, which is a value close to the YM of the normal tissue (background).

The mean values of PR for all treated and untreated mice at three different time points (week 1, week 2 and week 3) with the corresponding standard deviations are shown in Fig. 7(A2). At all time points, the treated mice were found to have higher PR than the untreated ones. For both the treated and untreated mice, the mean PR does not appear to change significantly at the different time points.

Figure 7(A3) shows the tumor/background YM contrast (CTYM) for the twelve treated and seven untreated cancers, while Fig. 7(A4) shows the tumor/background PR contrast (CTPR). In Fig. 7(A3), we see that the CTYM for untreated cancers are higher than that for the treated ones in all three weeks, which confirms previous observations^28^.

## Discussion

In this paper, we propose a new, non-invasive method for simultaneously reconstructing both the YM and PR in tumors. The YM is a mechanical parameter that has been investigated as a marker for cancer diagnosis, prognosis and treatment monitoring and planning. PR is another mechanical parameter, whose role in cancer assessment remains largely unexplored, even though mechanical testing data have demonstrated variability in PR values across different cancers^29,30^. To our knowledge, our proposed method is the first one to allow experimentally imaging the actual PR in cancers. From basic biomechanical theoretical considerations and as demonstrated with simulations and experiments in this study, estimation of PR is required for a correct estimation of the YM. Without an experimental method to assess the PR, an assumption about the PR value has to be made to compute the YM. It is a common assumption in many studies reported in the literature pertaining elastography to treat tumors and soft tissues as incompressible elastic solids^4–6^. Our study shows that, if not fully satisfied, such assumption can lead to significant errors in the reconstructed YM values (Fig. 1(A2) sample X4) even in the case of small YM tumor/background contrasts, and that this error increases as the YM tumor/background contrast increases. Thus, accurate estimation of the PR is not only important because of its potentials to provide new clinical information but also to obtain accurate estimates of the YM distribution. Furthermore, knowledge of both YM and PR is needed to correctly estimate tissue transport parameters in cancers, such as fluid pressure^31^ and vascular permeability^32^, which are known to be of great clinical significance.

Our proposed method to assess YM and PR is based on the seminal work by Eshelby. Although Eshelby’s theory for inclusions has been used in several fields, this theory has never been incorporated into theoretical formulations aiming at reconstructing the YM and PR of an inclusion embedded into another material. Thus, our proposed optimization formulation is the first method that utilizes Eshelby’s theory to solve this important mechanical problem and can have a far-reached impact in many engineering fields in addition to medicine, such as soil mechanics, non-destructive material characterization, biomaterials and, in general, in applications aimed at estimating mechanical properties of inclusions embedded in different materials.

The proposed method to assess YM and PR in tissues has many advantages compared to previously proposed reconstruction techniques, which are currently used in elastography. It can accurately reconstruct the YM and PR of a tumor for a wide range of tumor/background YM contrast, tumor’s shapes and in many complex boundary conditions. The proposed method is also robust to practical experimental conditions that may deviate from the ideal ones such as non-uniform loading and when the YM inside the tumor is heterogeneous. Thus, the proposed method has the potential to significantly improve the way the YM of tumors is currently imaged and quantified as well as to provide a new means to image and quantify the PR of tumors and normal tissues *in vivo*.

In this study, we have reported an extensive analysis of the error associated to the proposed method in the estimation of the YM and PR in a variety of sample geometries, mechanical properties and testing conditions. We found that YM and PR can be estimated with very high accuracy (>99% and >95%) when the inclusion is spherical or elliptic and data is noise-free (i.e., data from FEM) (Fig. 1(A1)). As the shape of the inclusion deviates from the spherical and elliptic geometry, the error in the YM and PR estimation increases. However the error remains below 13% even for cylindrical shaped inclusions. For noisy strain data^7^, the error increases and can be as high as 7% for the spherical inclusion. The error in the YM and PR estimation also increases in the case of large inclusion/background YM contrasts and in the case of inclusions softer than the background. However, in all cases considered in this study (29), the accuracy of the proposed method to estimate the YM and PR is found to be greater than 85% (Fig. 1).

In addition to simulations and controlled experiments, the proposed method was tested in a cancer animal model *in vivo*. Based on our preliminary *in vivo* animal results, YM in the untreated tumors was found to be increasing with time, whereas YM in the treated ones did not change significantly with time. In most cancers (both treated and untreated), PR was found to be higher in treated tumors in comparison to the untreated ones, but we did not observe any statistical changes in PR with cancer progression. We also found the PR to be higher in the tumor than in the soft tissue. The values of PR found in this study match well with those previously reported in the literature as estimated using invasive techniques^9,14–16,33^. However, more comprehensive studies are needed to confirm our observations and to elucidate the role of PR in cancer diagnosis, prognosis or treatment. The shape regularity index (solidity) and surface area of the tumors were also used to further characterize the *in vivo* results (section 27 of supplementary).

As biological tissues are poroelastic in nature, poroelastographic data needs to be acquired for prolonged time intervals so that the strains at steady state could be correctly estimated. At steady state, a poroelastic tissue behaves as a linear elastic solid. In the case of poroelastic materials, reconstruction of mechanical parameters using the transient strains may lead to incorrect YM and PR estimates. However, if the tissue can be assumed to behave as a linear elastic material (rather than a poroelastic material), there would be no need to collect data for prolonged time intervals, and our proposed method can be used to estimate the tissue elastic properties from knowledge of the instantaneous strains.

Our proposed method is inherently a three-dimensional method, where, ideally, the three normal components of the strain tensor (axial, lateral and elevational) are used to reconstruct the YM and PR in the tissue (see J1, J2 and J3 of Eq. 5). However, experimental estimation of the elevational strain component requires data acquisition along the elevational direction^21,22^. Currently, most clinical studies involving ultrasound imaging use linear arrays, which allow only planar data acquisition similarly to what we have reported in this manuscript. Acquisition of elevational data using these arrays is difficult to accomplish in free-hand elastography, in general, and particularly challenging in poroelastography, where temporal data acquisition is required. Therefore, implicit to this and most of the reconstruction procedures proposed in elastography is the assumption that the normal lateral and elevational components of the strain are identical. We have proved with extensive simulations and error analysis in this study that such assumption introduces small errors (<6%) in the YM and PR reconstructed using our proposed method. This was demonstrated with a number of three-dimensional samples with different geometry, covering most cases of practical interest for clinical elastography (see supplementary material (section 17-A)) and partially corroborated using controlled experiments and independent mechanical tests.

The main limitations of the proposed method are related to the assumptions required by the analytical model. In this paper, we have demonstrated that the proposed method is robust to local shape variations and produces accurate YM and PR estimates in a large number of inclusions of different shapes. In the past, numerical models have been applied in approaches to reconstruct the YM^34,35^. In terms of shape representation, the numerical 3-D model may be more accurate. However, a numerical 3-D model of the tumor would require additional data acquisition and would significantly increase the computation time. Furthermore, when multiple parameters are to be estimated, convergence problems typically arise in numerical solutions. Another limitation of the proposed method is that, theoretically, the model assumes the elastic properties inside the tissue to be uniform. The uniformity assumption is frequently undertaken in works related to cancer imaging and tumor mechanics^11,36,37^. It should be noted that our method reconstructs the YM and PR on a pixel-by-pixel basis, and we have proven that the method can still provide highly accurate YM and PR estimates even in the case of heterogeneous YM and PR distributions (Fig. 1(A4)). The estimation error increases as the heterogeneity of the mechanical properties increases. However, we found the error to be below 7%, even when the heterogeneity of the YM inside the tumor is 30%. Furthermore, although the YM and PR reconstruction formulations were derived for a sample with a single inclusion, we show in the supplementary material that the proposed theory can accurately reconstruct the YM and PR also in samples containing a number of inclusions, by reconstructing the YM and PR of each of the inclusions separately (see supplementary material (section 17-C)). Therefore, our results demonstrate that the proposed method provides local estimates of the elastic properties but accuracy may decrease when the mechanical properties inside the tissues are not uniform.

In our experiments, we used a linear array transducer with 6.6 MHz center frequency and 50% bandwidth. At higher frequencies, the resolution of the strain elastograms improves^38^, and the sensitivity of the resulting elastograms also increases^39^. This is also true for the strain estimation method used in this paper^7^. As a result, the sensitivity of the proposed method to the YM and PR is expected to increase at higher frequencies. With higher sensitivity, the presented method can be used to more accurately monitor changes in tumor mechanical properties over time. A more comprehensive study investigating the potential advantages of higher frequency/bandwidth ultrasound systems on the reconstructed parameters should be carried out in the future.

Computational complexity may also be considered as a limitation of the current implementation of the proposed method. As our method is based on an optimization technique, it is less efficient than some of the YM estimation methods available in the literature.

## Conclusions

In this paper, we have developed a novel reconstruction method based on the Eshelby’s theory for materials with an inclusion. Our proposed method can accurately estimate and image both the YM and the PR of tumors and surrounding tissue *in vivo*, can be used with tumors of different shapes and is robust to changes in the boundary conditions of the tumor environment. Simulations and controlled ultrasound elastography experiments demonstrate that the proposed method is capable of reconstructing YM and PR with high accuracy (>85% in most cases) in many experimental scenarios of clinical relevance. Based on the potential role of YM and PR as markers for cancer diagnosis, prognosis and treatment efficacy, the proposed method can have a significant impact in the assessment of cancers and, in general, in the field of elasticity cancer imaging.

## Materials and Methods

### Theory

The local stress and strain inside and outside an inclusion due to the remote stress have been determined by Eshelby^26^ using the superposition principle and Green’s function. The remote stress is the applied stress that creates a uniform stress over the entire background. This was done using a virtual experiment, which is summarized in Fig. S17. The four steps of Eshelby’s virtual experiment can be written mathematically in terms of the Green’s function of the elastic body. The strain and stress inside the inclusion can be written as^6,26^

1 $$\varepsilon ={\varepsilon }^{0}+S\,:{\varepsilon }^{\ast },$$

2 $$\sigma ={\sigma }^{0}+{C}^{0}\cdot [S-I]\,:{\varepsilon }^{\ast },$$

where *ε*^0^ is the remote strain, ε^*^ is the eigenstrain, *σ*^0^ is the remote stress, *C*^0^ is the stiffness tensor of the background, *I* is the identity tensor and *S* is the Eshelby’s tensor. *ε*^0^, ε^*^ and *σ*^0^ are vectors of three components (axial, lateral and elevational). The relationship between the remote stress *σ*^0^ and *ε*^0^ can be expressed as

3 $${\sigma }^{0}={C}^{0}\,:{\varepsilon }^{0}.$$

The Eshelby’s tensor *S* is a function of the geometry of the inclusion and the PR of the background. In Eqs. (1) and (2), the eigenstrain can be written as^27^ (eq. 22.13)

4 $${\varepsilon }^{\ast }={(S+A)}^{-1}\,:(-{\varepsilon }^{0}),\,where\,A={[C-{C}^{0}]}^{-1}\cdot {C}^{0}.$$

*C* is the stiffness tensor in the inclusion. The expression of *A* is relevant to our problem, which has been determined in eq. (10) in Section 9 of the supplementary material. Let us indicate ε^*^ in Eq. (1) as ${\varepsilon }_{1}^{\ast }$ and ^ε*^ in Eq. (4) as ${\varepsilon }_{2}^{\ast }$. In the expression of ${\varepsilon }_{1}^{\ast }$, only the Eshelby’s tensor *S* is involved. This requires knowledge of the tumor (inclusion) geometry and the PR of the normal tissue (background). In the expression of ${\varepsilon }_{2}^{\ast }$, the YM and PR of the tumor and normal tissues are involved. A cost function can be defined as

5 $$\begin{array}{c}J({E}_{i},{\nu }_{i})={({J}_{1}({E}_{i},{\nu }_{i}))}^{2}+{({J}_{2}({E}_{i},{\nu }_{i}))}^{2}+{({J}_{3}({E}_{i},{\nu }_{i}))}^{2},where\\ \,{J}_{1}({E}_{i},{\nu }_{i})={\varepsilon }_{1}^{\ast }(1)-{\varepsilon }_{2}^{\ast }(1),\,{J}_{2}({E}_{i},{\nu }_{i})\\ \,={\varepsilon }_{1}^{\ast }(2)-{\varepsilon }_{2}^{\ast }(2),{J}_{3}({E}_{i},{\nu }_{i})={\varepsilon }_{1}^{\ast }(3)-{\varepsilon }_{2}^{\ast }(3).\end{array}$$

and by minimizing this cost function *J*, we can obtain the YM (*E*_*i*_) and PR (*V*_*i*_) of the tumor. Let us assume that the geometry of the tumor is axisymmetric, i.e., the dimension of the tumor is same along lateral and elevational direction. In this case, the lateral and elevational components of strains are equal and we can write the cost function as

6 $$J({E}_{i},{\nu }_{i})={({J}_{1}({E}_{i},{\nu }_{i}))}^{2}+{({J}_{2}({E}_{i},{\nu }_{i}))}^{2}.$$

**Figure 8:**
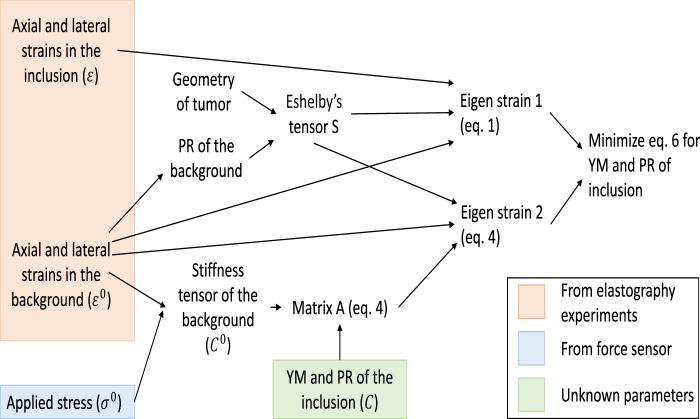
Flow diagram of the proposed method. All the known parameters are shown in square box.

The YM and PR of the normal tissue can be determined by using Eq. (3). The expressions of ${\varepsilon }_{1}^{\ast }$ and ${\varepsilon }_{2}^{\ast }$ for elliptic (prolate, oblate) and spherical tumor (inclusion) are shown in sections 10 and 14 of the supplementary material. The expressions of the Eshelby’s tensor *S* for cylindrical, flat elliptic, penny-shaped tumors are given in sections 11, 12 and 13 of the supplementary material. Using these *S* in equations of ${\varepsilon }_{1}^{\ast }$ and ${\varepsilon }_{2}^{\ast }$ for elliptic tumor, ${\varepsilon }_{1}^{\ast }$ and ${\varepsilon }_{2}^{\ast }$ for these shapes can be determined (and therefore YM and PR). The method has been shown in block diagram in Fig. 8.

### Controlled experiments

Eight non-homogenous phantoms were created with the background made of either hard tofu or extra-hard tofu (Morinaga Nutritional Foods, Inc., Torrance, CA USA) and the inclusion made of polyacrylamide^7,40^. The size of the background was 80 mm × 60 mm × 40 mm while the cylindrical inclusion diameter was 15 mm. The experiments were performed using our Sonix RP system (Ultrasonix, Richmond, BC, Canada) with a 38-mm linear array transducer, which operates with a center frequency of 6.6 MHz, bandwidth 5–14 MHz and beamwidth equal to 1 mm at the focus. Compression was applied from the top using different weights ranging 100 g to 400 g. A compressor plate was attached to the transducer face to apply the compression on a large area. A schematic of the setup for controlled experiments is shown in Fig. S26. The mechanical measurement procedure of the phantoms is described in section 26 of the supplementary material.

### *In vivo* experiments

Experiments on nineteen mice with triple negative breast cancer cells injected in the mammary fat pad were carried out on a weekly basis for three consecutive weeks. The cancers were created at the Houston Methodist Research Institute by injection of the cancerous cells beneath the mouse’s mammary fat pad^41^. *In vivo* data acquisition was approved by the Houston Methodist Research Institute, Institutional Animal Care and Use Committee (ACUC-approved protocol # AUP-0614-0033). All experiments were performed in accordance with relevant guidelines and regulations. Seven mice were kept untreated and twelve mice were treated by injecting them intravenously with one of the following drugs: 1. Epirubicin alone, 2. Liposomes loaded with Epirubicin and 3. Liposomes loaded with Epirubicin and conjugated with Lox antibody on the particle surface. The dose of each drug was 3 mg/kg body weight once a week. Prior to ultrasound data acquisition, each mouse was anesthetized with isoflurane. Each data acquisition session was 5 minutes long, and several RF data acquisitions could be performed during this period (for reliability purposes).

Elastography was carried out using a 38-mm linear array transducer (Sonix RP, Ultrasonix, Richmond, BC, Canada) with a center frequency of 6.6 MHz and 5–14 MHz bandwidth, 1 mm beamwidth at the focus. This acquisition configuration is commonly used for imaging lesions/tumors using ultrasound elastography in both proof of principle and clinical studies as shown by different labs^42,43^. To compensate for the surface geometry as well as facilitate positioning the focus inside the superficial tumors, an aqueous ultrasound gel pad (Aquaflex, Parker Laboratories, NJ, USA) was placed between the compressor plate and the developed tumor. It should be noted that such use of gel pad does not change the stress distribution inside the sample significantly and thus does not change the estimated parameters. This has been proved in section 7 of the supplementary. A force sensor (Tekscan FlexiForce) was inserted between the gel pad’s top surface and the compressor plate to record the applied force during the compression. Creep compression was performed manually on the animals and monitored using the force sensor, with the duration of each compression being one minute. Duration of the experiment was selected based on the temporal behavior of the soft tissue and tumor reported in the literature^44^ and to ensure that both the tumor and surrounding tissues reached steady state conditions. Ultrasound radio-frequency (RF) data acquisition was synchronized to the application of the compression. The sampling period of the data was set at 0.1 s. The axial and lateral strain data were calculated at steady state, when both the tumor and normal tissues behave as elastic materials^45^. An expert radiologist is employed to segment the *in vivo* axial strain elastograms in Matlab for determining the tumor areas. All the images from *in vivo* experiments shown are of size 4 cm (depth) by 3.8 cm (transducer length). Calculation procedure of stress from force sensor reading has been described in supplementary material (section 21).

### Simulation procedures

The FEM and ultrasound simulation methods are reported in sections 1 and 6 of the supplementary material. Details on the method to simulate different boundary conditions are given in section 2 of the supplementary material. The simulation methods used to create the heterogeneity of the YM distribution inside the tumor are discussed in section 3 of the supplementary material. Sections 4 and 5 of the supplementary material describe the procedure to simulate non uniform loading condition as well as samples with multiple layers above the inclusion. Specifications of the samples used in the simulations are reported in section 25 of the supplementary.

### Implementation of the proposed method and other computations

Details on the implementations of the proposed method, 3DB and 3DS are reported in the supplementary material (sections 22 and 23). Method used to compute the RMSE is described in section 24 of the supplementary material.

### Statistical analysis

Data in Figs. 7 and S29 are presented as mean ± SD (standard deviation). Matlab (MathWorks Inc., Natick, MA, USA) was used to statistically analyze the data. Statistical significance (99.9%, 99% and 95% confidence interval) was determined using the Kruskal-Wallis test.

## Supplementary information

Supplementary materials.

## Data Availability

The datasets generated during and/or analyzed during the current study are available from the corresponding author on reasonable request.
