# Supplementary material for: Non-invasive imaging of Young’s modulus and Poisson’s ratio in cancers in vivo
Source: Sci Rep. 2020 Apr 29;10:7266. doi: 10.1038/s41598-020-64162-6 (PMC7190860; doi:10.1038/s41598-020-64162-6)
Supplement: Supplementary file 1 — Supplementary materials. [file 41598_2020_64162_MOESM1_ESM.pdf]

Non-invasive imaging of Young's modulus and Poisson's ratio in cancers in vivo
Supplementary information

Md Tauhidul Islam, Songyuan Tang, Chiara Liverani, Sajib Saha, Ennio Tasciotti and Raffaella Righetti

1. Validation of the proposed approach by finite element analysis

Model. The poroelastic sample containing a poroelastic inclusion used for the analysis reported in this paper is shown in Fig. S1 (A). In this figure, we see that the sample is of cylindrical shape. The inclusion can be of different shape as considered in the paper. However, we assumed that the shape of the inclusion is always axisymmetric, i.e., the shape remains the same if a 2D plane containing the inclusion is revolved around the center line. As an example, we show a spherical inclusion of radius a inside the sample in Fig. S1 (A), which is perfectly axisymmetric. Because of the cylindrical symmetry of the sample and axisymmetry of the inclusion, the solution plane for this problem can be assumed as 2D as shown in Fig. S1 (B). From this figure, we also see that the compression is applied from the top and the bottom side is fixed. Two frictionless compressor plates have been used for holding up the sample and exert compression upon it.

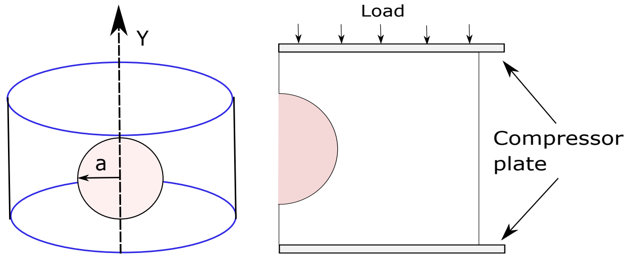

Fig. S1. (A) A schematic of a cylindrical sample of a poroelastic material with a spherical poroelastic inclusion of radius a. The axial direction is along the y-axis. (B) The 2D solution plane for the three dimensional sample. The sample is compressed between two compressor plates. The compression is applied along the negative y direction.

Finite element simulations. A commercial finite element simulation software namely Abaqus, Dassault Systemes Simulia Corp., Providence, RI, USA has been used for the finite element simulation study. Both the inclusion and background of the sample are modeled as a linearly elastic, isotropic, incompressible, permeable solid phase saturated with an incompressible fluid.

The sample is compressed from the top and the bottom side is kept static. An instantaneous load of 1000 Pa is applied and kept constant for a certain time interval. This value of compression is chosen based on elastography experiments. The mesh used to model the sample is CAX4RP and has $81,790$ elements in the solution plane. A zero fluid pressure boundary condition on the right hand side of the samples is imposed to ensure the flow of the fluid only in the right direction. We refer to Islam et al. (1) for details of the poroelastic simulation in Abaqus. The dimension of the solution plane of the sample is 2 cm in radius and 4 cm in height.

In all simulated samples, the interstitial permeability of the inclusion is taken as 3.1×10^{-14} m^2 (Pa s)$^{-1}$ and of the background is taken as 6.4×10^{-15} m^2 (Pa s)$^{-1}$. The vascular permeability of the inclusion is assumed 5.67×10^{-7} (Pa s)$^{-1}$ and of the background is assumed 1.89×10^{-8} (Pa s)$^{-1}$. These values of material properties for the inclusion and background are chosen following Leiderman et al. (2). Void ratio in all the samples is assumed equal to 0.4. These material properties are kept the same in all the samples (A-M, Z1-Z8, X1-X9, H1-H3, B1-B3 and R1-R4) while the values of YM and PR are varied to create different simulation conditions. In the samples that have non-uniform background, i.e., small inclusions, stiff strip of tissue, etc. the values of interstitial and vascular permeabilities for these objects in the background are assumed the same as those for the background. The analysis time in Abaqus is chosen for different samples in such a way that the samples reach steady state and behave as fully linear elastic materials at the end of the analysis. The axial and lateral strains used in all the YM and PR reconstructions reported in the paper are computed at the steady state.

For segmenting the steady state axial and lateral stain elastograms from finite element analysis (FEA), a morphological segmentation algorithm is used (3).

Reconstruction of YM and PR from FEA data of samples A-M. The radius of the spherical inclusion in samples A-M is 0.3 cm. The mechanical parameters of samples A-M are tabulated in Table S1. In this table, E_b and E_i denote the YM of the normal tissue (background) and tumor (inclusion) and ν_b and ν_i denote their PR.

Table S1. YM and PR of samples A-M used in the FEA and ultrasound simulations

Sample name	E_b (kPa)	E_i (kPa)	ν_b	ν_i
A	32.78	97.02	0.49	0.40
B	32.78	50.00	0.49	0.40
C	32.78	163.90	0.49	0.40
D	32.78	97.02	0.45	0.45
E	32.78	97.02	0.20	0.45
F	32.78	97.02	0.20	0.30
G	32.78	491.70	0.20	0.45
H	32.08	819.50	0.20	0.45
I	32.78	1639.00	0.20	0.45
J	32.78	3278.00	0.20	0.45
K	32.08	16.39	0.20	0.30
L	32.78	6.556	0.20	0.30
M	32.78	3.278	0.20	0.30

The steady state axial and lateral strains for the first four samples (A-D) from FEA are shown in Figs. S2 (A1-A4) and (B1-B4), respectively. In this figures, we see that the axial and lateral strains are constant inside the inclusion, which correlates with Eshelby's theory (4).

The reconstructed YM of samples A-D by the proposed method and the other two methods used for the comparison are shown in Fig. S3 (A1-A4), (B1-B4) and (C1-C4), respectively. The reconstructed PR of samples A-D by the proposed method are shown in Fig. S4 (A1-A4).

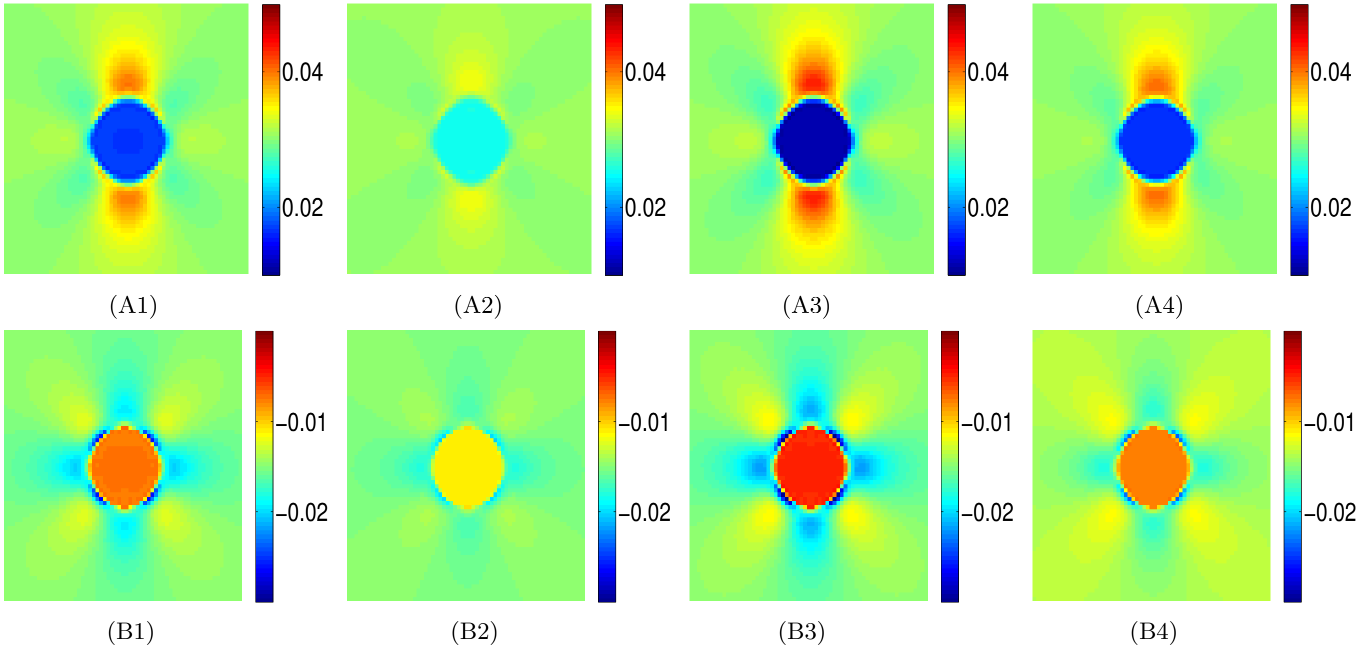

Fig. S2. (A1)-(A4) Axial strains from finite element simulation for samples A-D (B1)-(B4) Lateral strains from finite element simulation for samples A-D.

Specifications of the samples Z1-Z8 and X1-X9. For samples Z1-Z8, the YM and PR of tumor are set to 97.02 kPa and 0.3, whereas the YM and PR of the normal tissue are set to 32.78 kPa and 0.2. For samples X1-X9, the YM and PR of tumor are set to 97.02 kPa and 0.45 and the YM and PR of the normal tissue are set to 32.78 kPa and 0.2.

All the samples simulated are of 4 cm height and 2 cm width in an axisymmetric setup. In samples X1-X9, the radius of the spherical inclusion is 0.3 cm. In sample Z1, the radius of the inclusion is 0.3 cm, the lengths of elliptical axes along lateral and axial direction in inclusions of samples Z2 and Z3 are 0.2 cm and 0.5 cm and 0.5 cm and 0.2 cm, respectively. The radius and height of the cylindrical inclusion of sample Z4 are 0.3 cm and 0.55 cm. The radius of the penny-shaped inclusion of sample Z5 is 0.5 cm and the height is 0.05 cm. The length of each side of tetragonal, pentagonal and hexagonal inclusions in samples Z6, Z7 and Z8 are 0.45 cm.

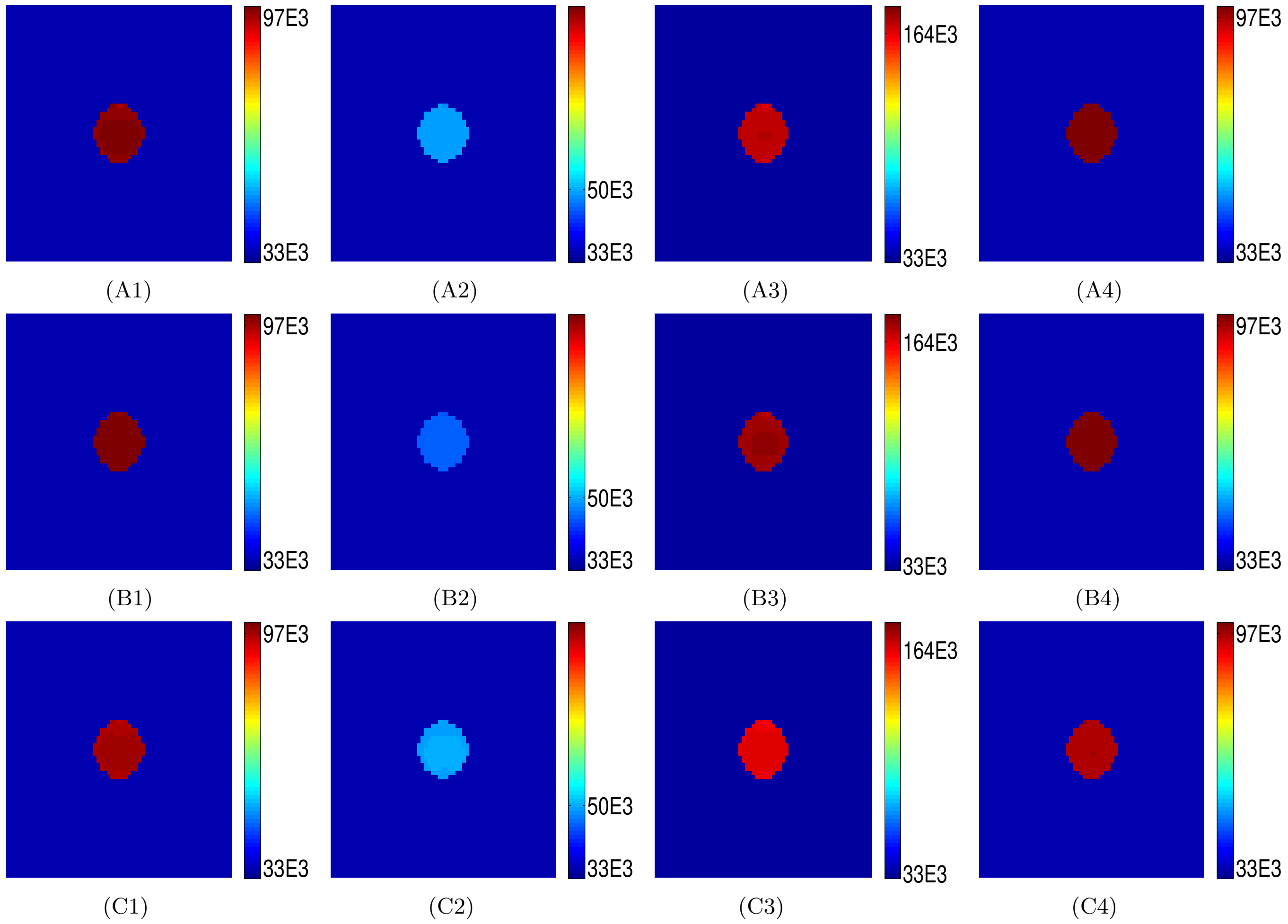

Fig. S3. (A1)-(A4) Reconstructed YM of samples A-D by the 3DB approach and (B1)-(B4) reconstructed YM of samples A-D by the 3DS approach from FEA axial strain data. (C1)-(C4) Reconstructed YM of samples A-D by the proposed approach from FEA axial and lateral strain data.

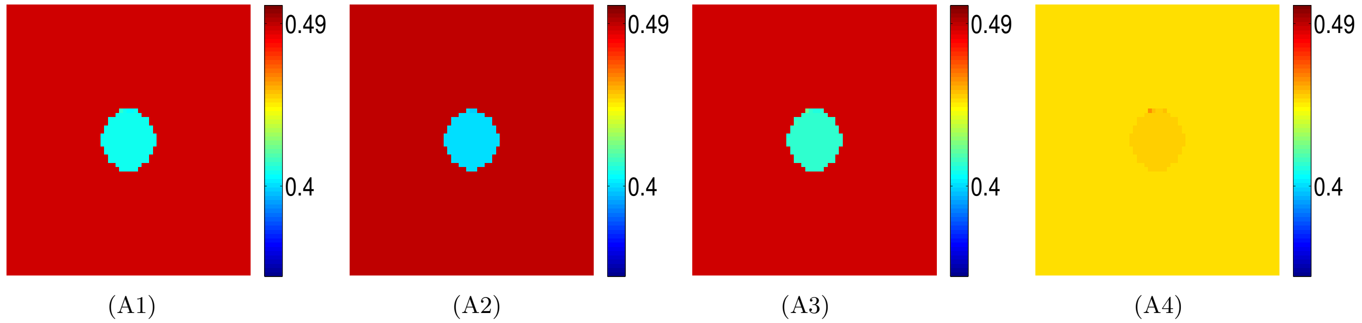

Fig. S4. (A1)-(A4) Reconstructed PR of samples A-D by the proposed approach from FEA axial and lateral strain data.

2. Finite element models of samples with complex boundary conditions

A. Samples with zig-zag stiff tissue, spheres and ellipses in the background. We have chosen two samples B1 and B2 with two different complex boundary conditions. In sample B1, we simulate zig-zag stiff materials (YM of 40 kPa) in the normal tissue region. In sample B2, there are fourteen spherical and elliptical inclusions in the normal tissue region with different YM (45 kPa, 50 kPa and 60 kPa) and PR (0.15, 0.2, 0.22, 0.25 and 0.3). The YM in the inclusion and normal tissue is 97.02 and 32.78 kPa, respectively in sample B1 and B2. In both samples, the main inclusion has a radius of 1 cm. The lowest distance from the stress application plane to the inclusion is 1 cm. As shown in the FEA model (Fig. S5), the total area of the sample considered is 8 cm× 4 cm. This area is chosen based on the size of commonly used gelpad (9 cm diameter) and compressor plate (10.1 cm × 9.3 cm) and the depth of ultrasound penetration used in common experiments which is 4 cm. The imaging region is the center square portion of 4 cm× 4 cm.

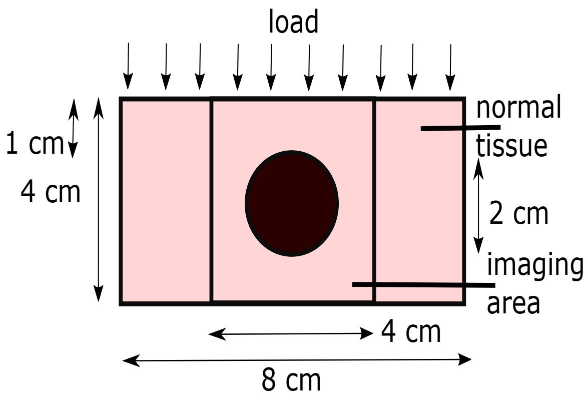

Fig. S5. Finite element model to investigate the impact of complex boundary conditions on the estimation of YM and PR by the proposed method.

B. Sample with a strip of stiff tissue above the tumor. The finite element model of the sample B3 with a strip of stiff tissue above the tumor is shown in Fig. S6. The YM in the tumor is 97.02 kPa and in the normal tissue is 32.78 kPa. YM in the strip stiff tissue is 50 kPa. PR in the tumor is assumed as 0.3 and in the normal tissue and stiff tissue strip as 0.2. The radius of the spherical inclusion inside sample B3 is 0.3 cm. The height and width of sample B3 are 4 cm and 2 cm, respectively.

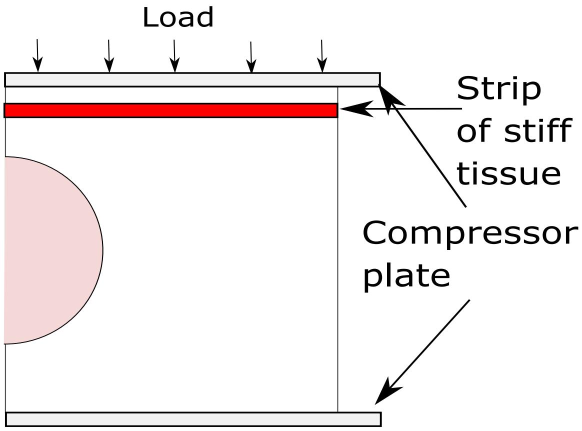

Fig. S6. Finite element model of samples B3 with a strip of stiff tissue above the spherical tumor.

3. Samples with a heterogeneous YM distribution inside the tumor

The finite element models for samples H1, H2 and H3 with heterogeneous YM distributions inside the tumor are shown in Fig. S7. The inclusion in samples H1-H3 is made up of three concentric spheres of outer radii 0.1, 0.2 and 0.3 cm. The inner sphere has YM modulus of 97.02 kPa in samples H1-H3 and the next spherical shell has YM of 92.17 kPa for 10% heterogeneity (sample H1), 87.32 kPa for 20% (sample H2) and 82.46 kPa for 30% heterogeneity (sample H3). The peripheral spherical shell has YM of 87.32 kPa for 10% heterogeneity, 77.62 kPa for 20% and 67.91 kPa for 30% heterogeneity. The YM of the normal tissue is assumed to be 32.78 kPa. The PR of the tumor is taken as 0.3 and of the normal tissue is taken as 0.2. The height and width of samples H1-H3 are 4 cm and 2 cm, respectively.

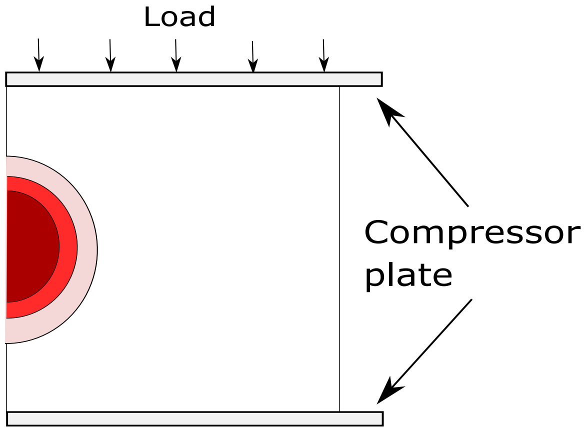

Fig. S7. Finite element model of samples H1, H2 and H3 with heterogeneous YM distribution inside the tumor.

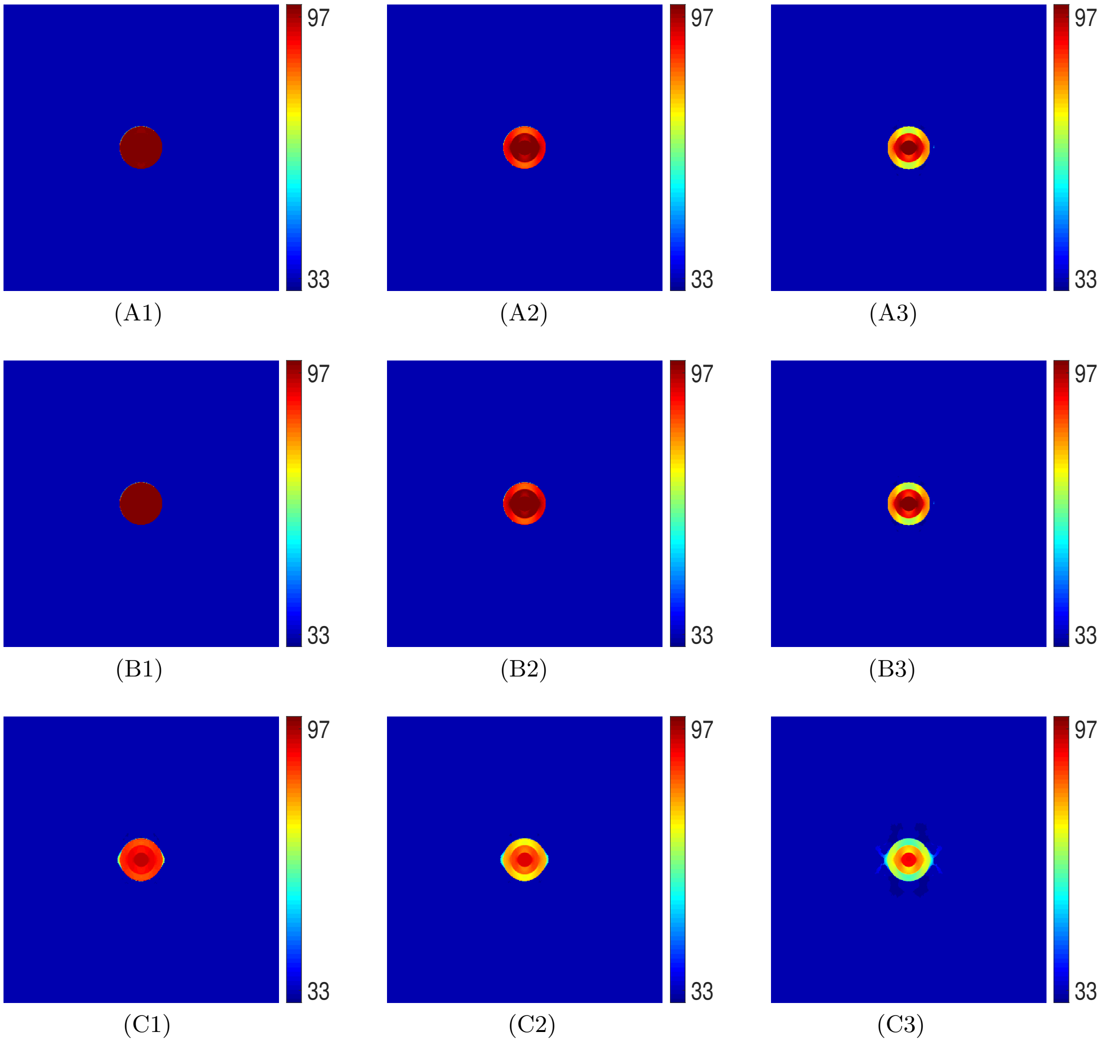

Fig. S8. (A1)-(A3) Reconstructed YM (in kPa) of samples H1-H3 by the 3DB approach and (B1)-(B3) reconstructed YM (in kPa) of samples H1-H3 by the 3DS approach from FEA axial strain data. (C1)-(C3) Reconstructed YM (in kPa) of samples H1-H3 by the proposed approach from FEA axial and lateral strain data.

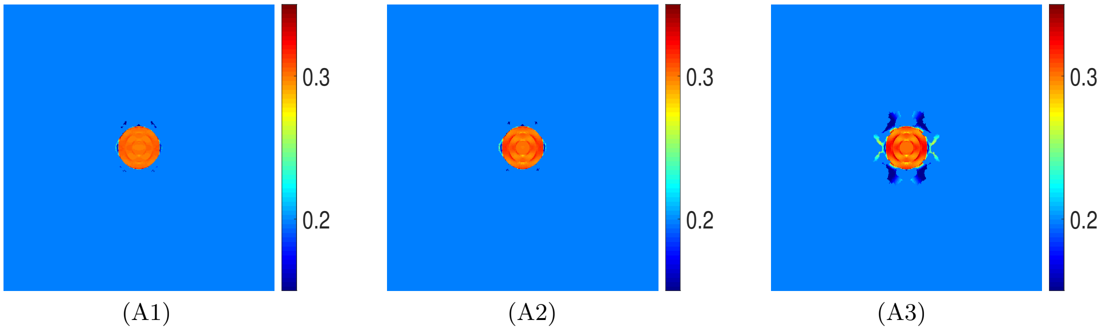

Fig. S9. (A1)-(A4) Reconstructed PR of samples H1-H3 by the proposed approach from FEA axial and lateral strain data.

4. Finite element model of samples subjected to non-uniform compression

Among the simulated samples subjected to non-uniform compressions (shown in Fig. S10) in FEA, in sample R1, the load is 1 kPa in the center, which gets reduced by 20% at the side of the imaging area. In sample R2, the load is 1 kPa in the center, which gets reduced by 10% at the side of the imaging area. In sample R3, the load is 1 kPa in the center which increases by 20% at the side of the imaging area. In sample R4, the load is 1 kPa in the center which increases by 10% at the side of the imaging area. In samples R1-R4, the YM and PR of the inclusion are set to 97.02 kPa and 0.3, whereas the YM and PR of the background are set to 32.78 kPa and 0.2.

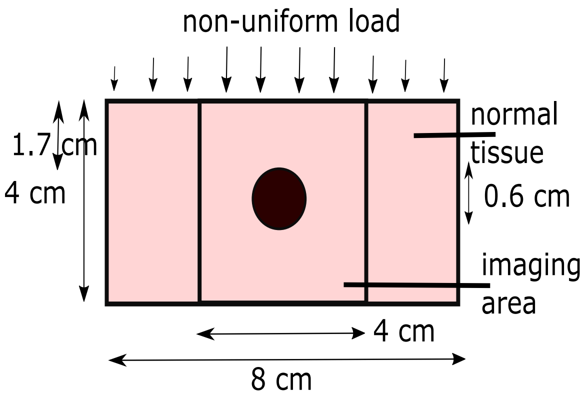

Fig. S10. Finite element model to investigate the impact of non-uniform compression on the estimation of YM and PR by the proposed method.

5. Finite element model of samples with multiple layers above the inclusion

The FEA model for samples with multiple layers of soft tissue with different stiffness is shown in Fig. S11. We chose the YM of the four layers as 30 kPa, 49.17 kPa, 65.56 kPa and 81.95 kPa in sample B4 and B5. We selected 32.78 kPa as the YM of the background tissue. In both the layer tissues and normal tissues, we assumed PR of 0.4 in sample B4, whereas we assumed PR of 0.25 in sample B5. The YM of the tumor has been assumed 97.02 and 163.90 kPa in sample B4 and B5, respectively. The PR of the tumor has been assumed as 0.4 in sample B4 and 0.3 in sample B5.

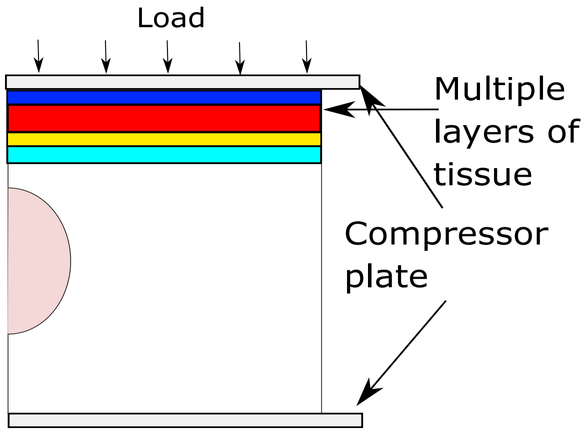

Fig. S11. Finite element model of samples B4-B5 with four layers of tissue of different YM above the spherical tumor.

6. Ultrasound simulation

Simulation method. The simulated pre- and post-compression ultrasound RF data are generated from the mechanical displacements using a convolution model (5). Bilinear interpolation is performed on the input mechanical displacement data (obtained from FEA) prior to the computation of the simulated RF frames (6). The simulated ultrasound transducer has 128 elements, frequency bandwidth between $5-14$ MHz, a 6.6 MHz center frequency, and 50% fractional bandwidth at -6 dB. The transducer's beamwidth is assumed to be dependent on the wavelength and to be approximately 1 mm at 6.6 MHz (7). The sampling frequency is set at 40 MHz and Gaussian noise is added to set the SNR at 40 dB. From the same sets of simulated pre- and post-compression RF data, the method proposed by Islam et al. (8) is used to estimate the axial and lateral strains. Axial and lateral strains from 50 independent realizations are averaged to obtain the final axial and lateral strains.

For segmenting the axial and lateral stain elastograms from ultrasound simulated data, a morphological segmentation algorithm is used as in (3).

Comparison. The axial and lateral strains for ultrasound simulations for the first four samples (A-D) are shown in (A1)-(A4) and (B1-B4) of Fig. S12.

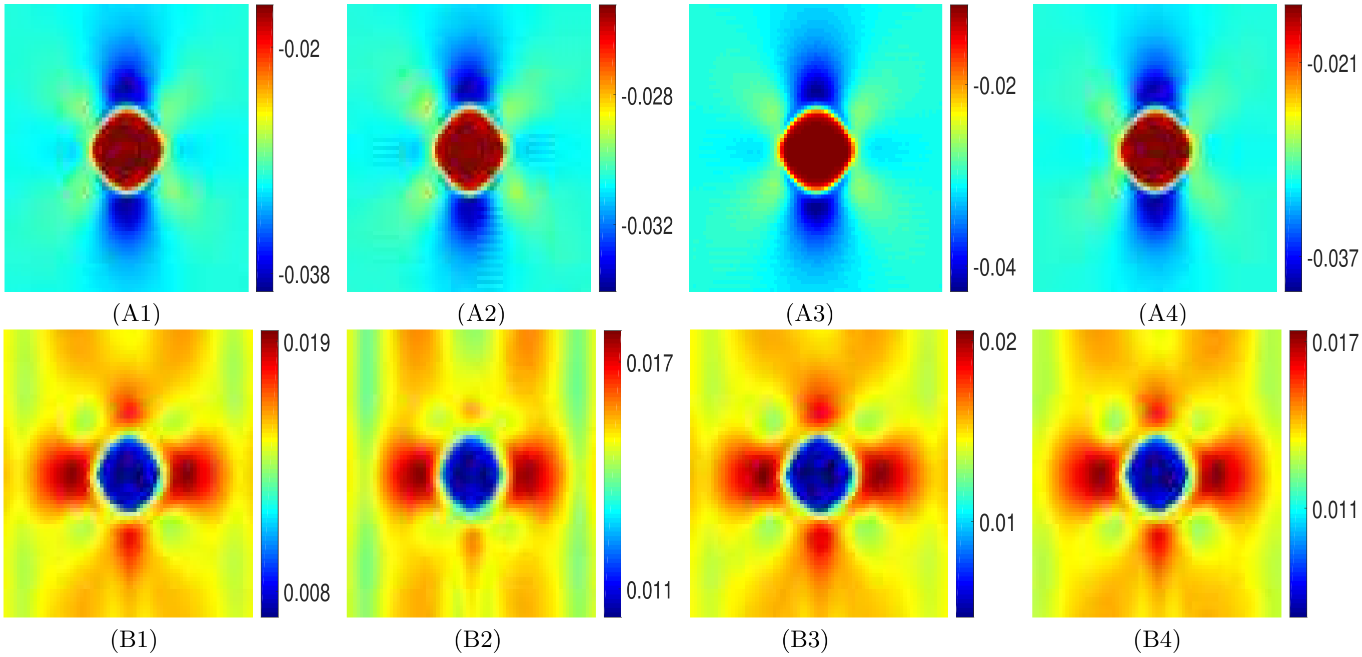

Fig. S12. (A1)-(A4) Estimated axial strains from ultrasound simulation data for samples A-D (B1)-(B4) estimated lateral strains from ultrasound simulation data for samples A-D.

The estimated values of YM by the 3DB approach are shown in (A1-A4) of Fig. S13 for ultrasound simulated RF data of samples A-D. The estimated values of YM appear uniform inside the inclusion, but the values significantly deviate from the true values. The reconstructed YM by the 3DS approach are shown in (B1-B4) of Fig. S13 for ultrasound simulated RF data of samples A-D. The reconstructed YM and PR by our proposed approach are shown in (C1-C4) of Fig. S13 and (A1-A4) of Fig. S14. The estimated YM and PR by our technique are closer to the true values than the estimated YM and PR estimated by other two methods.

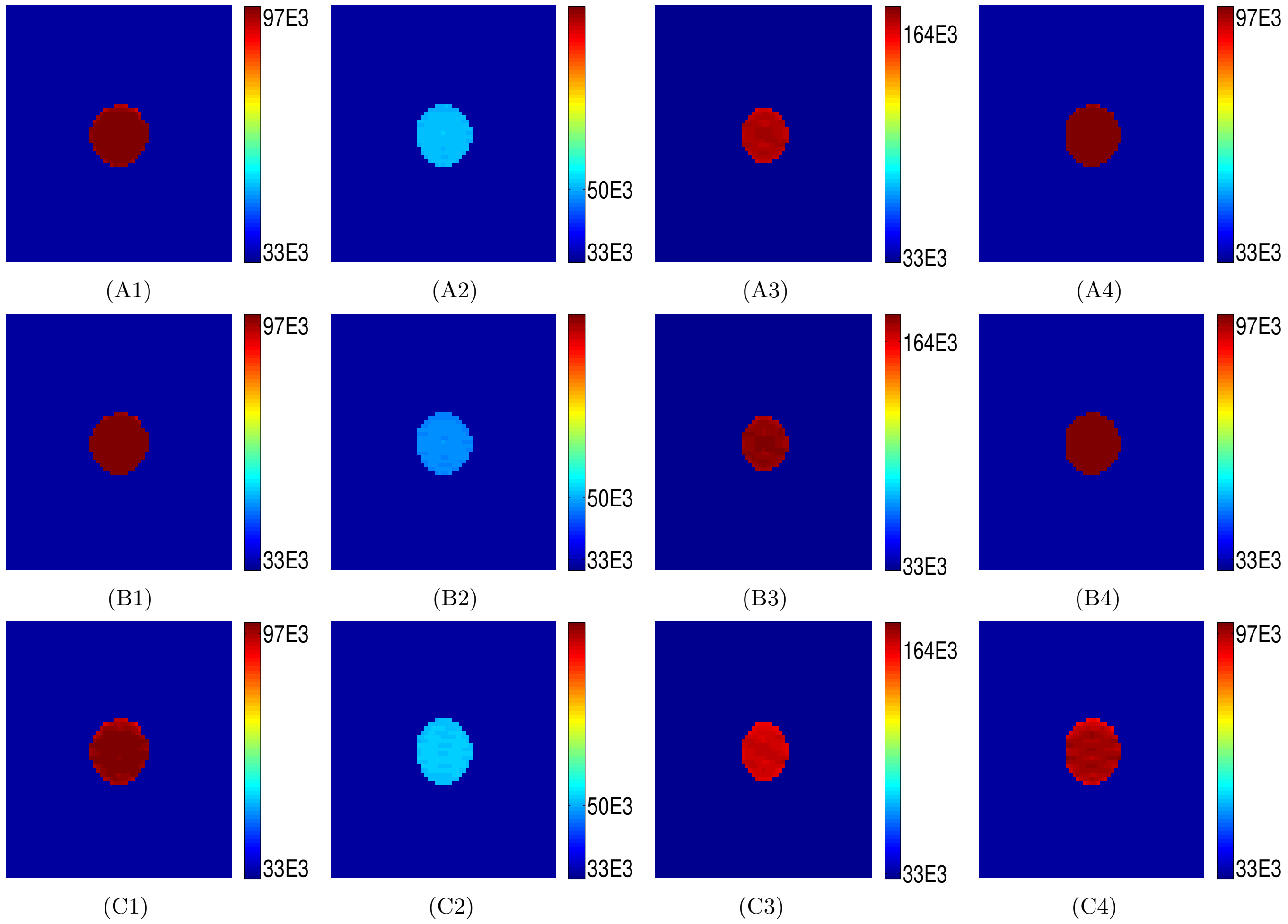

Fig. S13. (A1)-(A4) Reconstructed YM in samples A-D by the 3DB approach as applied to ultrasound simulated RF data. (B1)-(B4) Reconstructed YM in samples A-D by the 3DS approach and (C1)-(C4) reconstructed YM in samples A-D by the proposed method as applied to ultrasound simulated RF data.

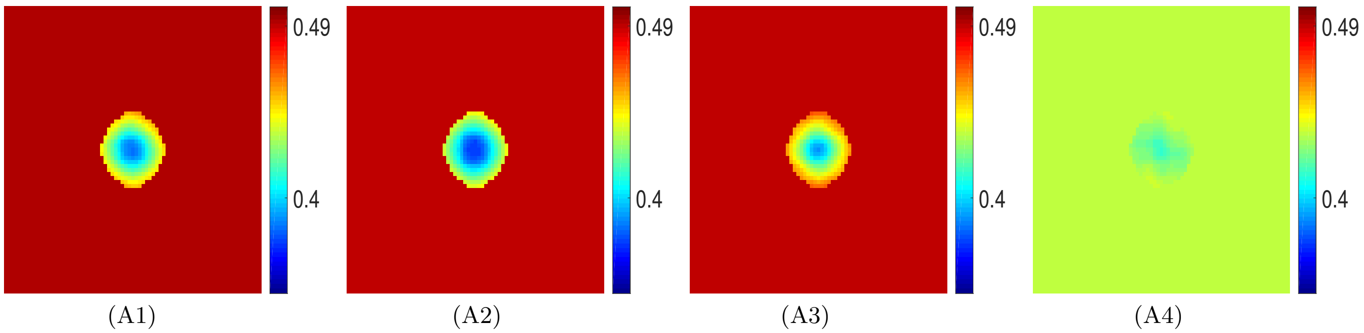

Fig. S14. (A1)-(A4) Reconstructed PR in samples A-D using the proposed approach as applied to ultrasound simulated RF data.

7. Stress distribution in samples with gel pad

The finite element model for simulating the stress and strain distributions in samples with a gel pad at the top is shown in Fig. S15. The YM and PR of normal tissue have been assumed 32.78 kPa and 0.4. The YM and PR of the tumor have been assumed 97.02 kPa and 0.4. The YM of the soft gelpad has been assumed 16.39 kPa and of the stiff gelpad has been assumed 65.56 kPa. The PR of both type of gelpad has been assumed 0.4. A load of 1 kPa has been applied on the top surface in this model. The stress distributions for the stiff and soft gel pads are shown in Fig. S16. We show the mean axial and lateral stresses in all three cases in Table S2. We see from this table that the axial and lateral stresses change insignificantly because of the gelpad usage. Based on these simulation results, it can be said that the observed strains in the tissues in our elastography experiments are not affected by the presence of the gelpad.

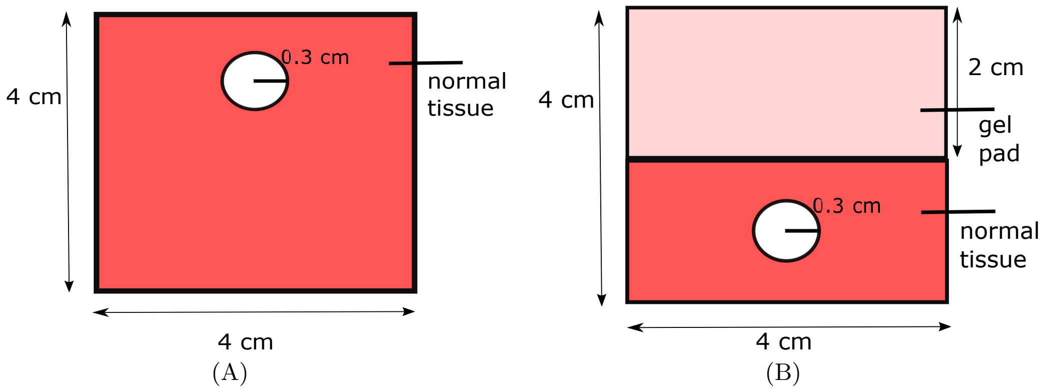

Fig. S15. Finite element model of a non-uniform sample with gelpad at the top used to investigate the impact of the use of gelpad on the observed strains in our in vivo tissues. Sample (A) without gelpad (B) with gelpad.

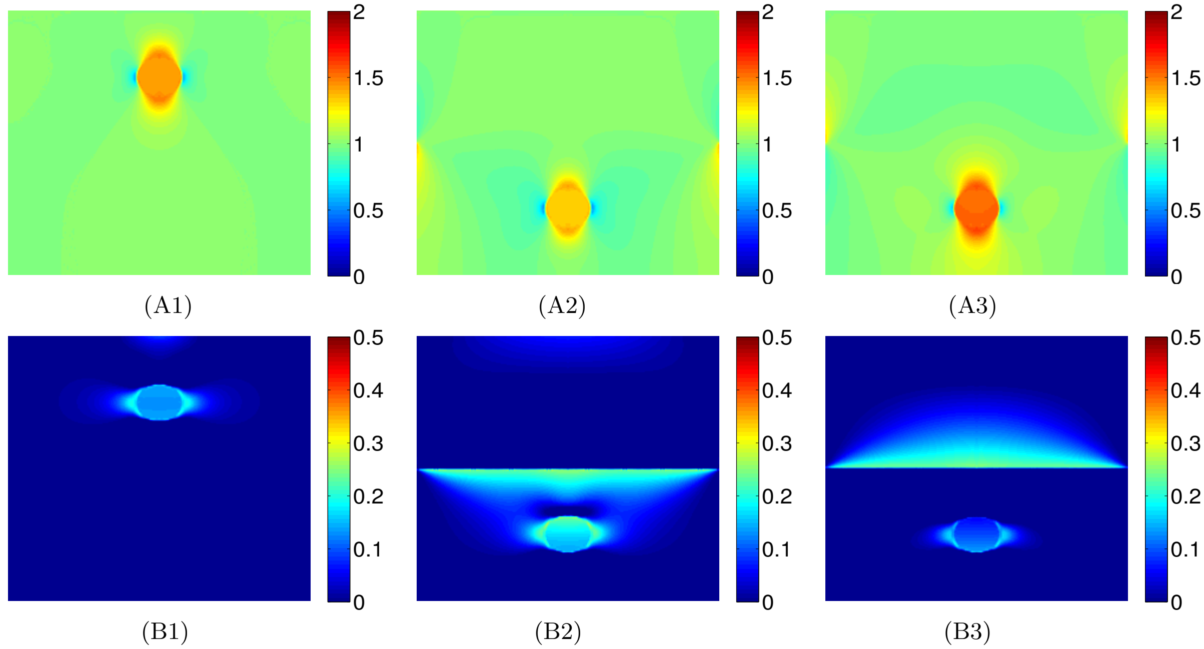

Fig. S16. Axial stress (A1) without gelpad (A2) with 50% softer and (A3) 200% stiffer gelpad with respect to normal tissue. Lateral stress (B1) without gelpad (B2) with 50% softer and (B3) 200% stiffer gelpad with respect to normal tissue.

8. Eshelby's virtual experiment

The virtual experiment is composed by the following steps:

1. Isolate the inclusion from the background (Fig. S17(A)). Consequently, the inclusion is strained because of the loss of constraint imposed by the background. This strain is denoted as eigenstrain (ϵ^*).

2. Apply traction T to bring the inclusion in its original shape (Fig. S17(B)). The strain induced inside the inclusion should compensate the eigenstrain.

Table S2. Mean axial and lateral stresses inside the inclusions for samples with and without gelpad

	Axial stress (kPa)	Lateral stress (kPa)
Sample without gelpad	1.43	0.13
Sample with soft gelpad	1.33	0.16
Sample with stiff gelpad	1.53	0.09

3. Insert the inclusion back in the background (Fig. S17(C)). The traction force is still T.
4. Remove the applied traction T (Fig. S17(D)). This is the same scenario as step 1 (Fig. S17(A)). The removal of the traction force from step 3 to step 4 is equivalent to applying a body force of $-T$ to the surface of the inclusion.

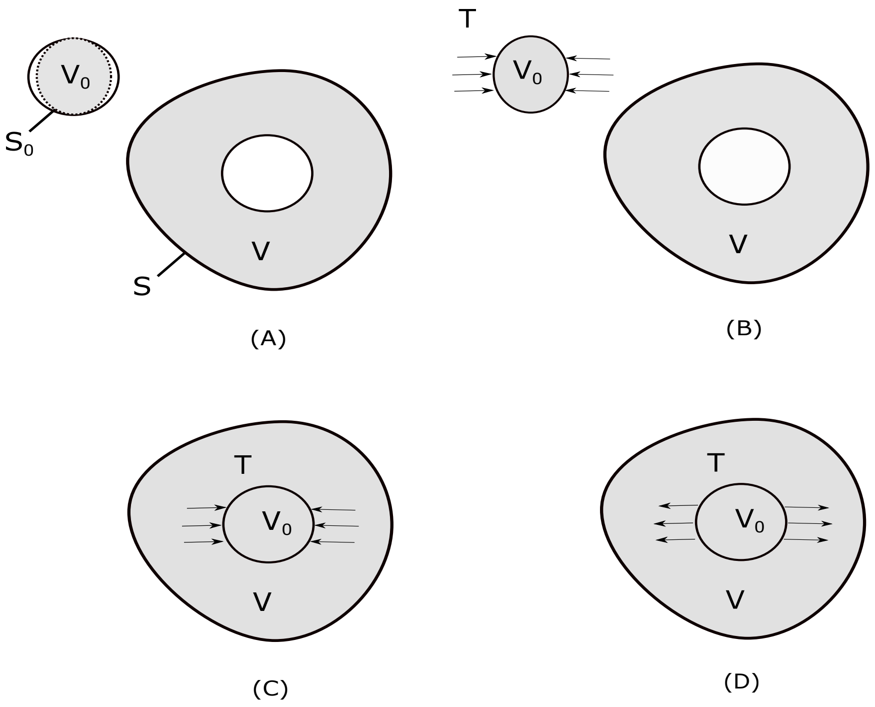

Fig. S17. Four steps of Eshelby's virtual experiment to reach the solution. Here, background is a linear elastic solid of volume V and surface S. The inclusion is also a linear elastic solid of volume V_0 and surface S_0. (A) The inclusion is removed from the background. (B) A surface traction T is applied to return V_0 in its original shape. (C) We put the inclusion back to the matrix and (D) remove the applied traction.

9. Expression of A

The stiffness matrix for the inclusion can be written as

$$\boldsymbol{C} = \begin{bmatrix} \lambda_i + 2\mu_i & \lambda_i & \lambda_i & 0 & 0 & 0 \\ \lambda_i & \lambda_i + 2\mu_i & \lambda_i & 0 & 0 & 0 \\ \lambda_i & \lambda_i & \lambda_i + 2\mu_i & 0 & 0 & 0 \\ 0 & 0 & 0 & \mu_i & 0 & 0 \\ 0 & 0 & 0 & 0 & \mu_i & 0 \\ 0 & 0 & 0 & 0 & 0 & \mu_i \end{bmatrix}, \tag{1}$$

where

$$\lambda_i = \frac{E_i \nu_i}{(1+\nu_i)(1-2\nu_i)}, \tag{2}$$

$$\mu_i = \frac{E_i}{2(1+\nu_i)}. \tag{3}$$

Here E_i and ν_i are the YM and PR of the inclusion. The stiffness matrix for the background can be written as

$$\boldsymbol{C^0} = \begin{bmatrix} \lambda_b + 2\mu_b & \lambda_b & \lambda_b & 0 & 0 & 0 \\ \lambda_b & \lambda_b + 2\mu_b & \lambda_b & 0 & 0 & 0 \\ \lambda_b & \lambda_b & \lambda_b + 2\mu_b & 0 & 0 & 0 \\ 0 & 0 & 0 & \mu_b & 0 & 0 \\ 0 & 0 & 0 & 0 & \mu_b & 0 \\ 0 & 0 & 0 & 0 & 0 & \mu_b \end{bmatrix}, \tag{4}$$

where

$$\lambda_b = \frac{E_b \nu_b}{(1+\nu_b)(1-2\nu_b)}, \tag{5}$$

$$\mu_b = \frac{E_b}{2(1+\nu_b)}. \tag{6}$$

Here E_b and ν_b are the YM and PR of the background.

If we assume that $\boldsymbol{X} = (\boldsymbol{C} - \boldsymbol{C^0})^{-1}$, then $\boldsymbol{X}$ can be written as

$$\boldsymbol{X} = \begin{bmatrix} \Psi & \Omega & \Omega & 0 & 0 & 0 \\ \Omega & \Psi & \Omega & 0 & 0 & 0 \\ \Omega & \Omega & \Psi & 0 & 0 & 0 \\ 0 & 0 & 0 & -1/(\mu_b - \mu_i) & 0 & 0 \\ 0 & 0 & 0 & 0 & -1/(\mu_b - \mu_i) & 0 \\ 0 & 0 & 0 & 0 & 0 & -1/(\mu_b - \mu_i) \end{bmatrix}, \tag{7}$$

where

$$\Psi = -(\lambda_b - \lambda_i + \mu_b - \mu_i)/(2\mu_b^2 + 2\mu_i^2 + 3\lambda_b\mu_b - 3\lambda_b\mu_i - 3\lambda_i\mu_b + 3\lambda_i\mu_i - 4\mu_b\mu_i), \tag{8}$$

$$\Omega = (\lambda_b - \lambda_i)/(2(2\mu_b^2 + 2\mu_i^2 + 3\lambda_b\mu_b - 3\lambda_b\mu_i - 3\lambda_i\mu_b + 3\lambda_i\mu_i - 4\mu_b\mu_i)). \tag{9}$$

The matrix $\boldsymbol{A} = [\boldsymbol{C} - \boldsymbol{C^0}]^{-1} \cdot \boldsymbol{C^0}$ can be written as

$$\boldsymbol{A} = \begin{bmatrix} \Psi(\lambda_b + 2\mu_b) + 2\lambda_b\Omega & \Omega(\lambda_b + 2\mu_b) + \lambda_b\Omega + \lambda_b\Psi & \Omega(\lambda_b + 2\mu_b) + \lambda_b\Omega + \lambda_b\Psi & 0 & 0 & 0 \\ \Omega(\lambda_b + 2\mu_b) + \lambda_b\Omega + \lambda_b\Psi & \Psi(\lambda_b + 2\mu_b) + 2\lambda_b\Omega & \Omega(\lambda_b + 2\mu_b) + \lambda_b\Omega + \lambda_b\Psi & 0 & 0 & 0 \\ \Omega(\lambda_b + 2\mu_b) + \lambda_b\Omega + \lambda_b\Psi & \Omega(\lambda_b + 2\mu_b) + \lambda_b\Omega + \lambda_b\Psi & \Psi(\lambda_b + 2\mu_b) + 2\lambda_b\Omega & 0 & 0 & 0 \\ 0 & 0 & 0 & \mu_b\Phi & 0 & 0 \\ 0 & 0 & 0 & 0 & \mu_b\Phi & 0 \\ 0 & 0 & 0 & 0 & 0 & \mu_b\Phi \end{bmatrix}, \tag{10}$$

where

$$\Phi = -\frac{1}{\mu_b - \mu_i}. \tag{11}$$

10. Expressions of ϵ_1^* and ϵ_2^* for elliptic inclusion

The Eshelby's tensor $\boldsymbol{S}$ can be written for an elliptic inclusion with semi-axis lengths of a, b and c along x, y and z-direction as (9)

$$\boldsymbol{S} = \begin{bmatrix} \boldsymbol{S}_{1111} & \boldsymbol{S}_{1122} & \boldsymbol{S}_{1133} & 0 & 0 & 0 \\ \boldsymbol{S}_{2211} & \boldsymbol{S}_{2222} & \boldsymbol{S}_{2233} & 0 & 0 & 0 \\ \boldsymbol{S}_{3311} & \boldsymbol{S}_{3322} & \boldsymbol{S}_{3333} & 0 & 0 & 0 \\ 0 & 0 & 0 & \boldsymbol{S}_{2323} & 0 & 0 \\ 0 & 0 & 0 & 0 & \boldsymbol{S}_{3131} & 0 \\ 0 & 0 & 0 & 0 & 0 & \boldsymbol{S}_{1212} \end{bmatrix}, \quad [12]$$

where

$$\boldsymbol{S}_{1111} = \frac{3}{8\pi(1-\nu_b)} a^2 I_{11} + \frac{1-2\nu_b}{8\pi(1-\nu_b)} I_1, \quad [13]$$

$$\boldsymbol{S}_{1122} = \frac{1}{8\pi(1-\nu_b)} b^2 I_{12} + \frac{1-2\nu_b}{8\pi(1-\nu_b)} I_1, \quad [14]$$

$$\boldsymbol{S}_{1133} = \frac{1}{8\pi(1-\nu_b)} c^2 I_{13} + \frac{1-2\nu_b}{8\pi(1-\nu_b)} I_1, \quad [15]$$

$$\boldsymbol{S}_{1212} = \frac{a^2+b^2}{16\pi(1-\nu_b)} a^2 I_{12} + \frac{1-2\nu_b}{16\pi(1-\nu_b)} (I_1 + I_2). \quad [16]$$

The other nonzero terms can be found by cyclic permutation of the above formulas. We have to let $a \to b \to c$ together with $1 \to 2 \to 3$.

Assuming $a > b > c$, the I-terms can be calculated as

$$I_1 = \frac{4\pi abc}{(a^2-b^2)(a^2-c^2)^{\frac{1}{2}}} \big[\chi(\theta,k) - \zeta(\theta,k)\big], \quad [17]$$

$$I_3 = \frac{4\pi abc}{(b^2-c^2)(a^2-c^2)^{\frac{1}{2}}} \big[\frac{b(a^2-c^2)^{\frac{1}{2}}}{ac} - \zeta(\theta,k)\big], \quad [18]$$

where

$$\theta = \sin^{-1}\sqrt{\frac{a^2-c^2}{a^2}}, \quad [19]$$

$$k = \sqrt{\frac{a^2-b^2}{a^2-c^2}} \quad [20]$$

and

$$I_1 + I_2 + I_3 = 4\pi, \quad [21]$$

$$3I_{11} + I_{12} + I_{13} = \frac{4\pi}{a^2}, \quad [22]$$

$$3a^2 I_{11} + b^2 I_{12} + c^2 I_{13} = 3I_1, \quad [23]$$

$$I_{12} = \frac{I_2 - I_1}{a^2 - b^2}. \quad [24]$$

The standard elliptic integrals χ and ζ are defined as

$$\chi(\theta,k) = \int_0^\theta \frac{dw}{\sqrt{1-k^2 sin^2 w}}, \quad [25]$$

$$\zeta(\theta,k) = \int_0^\theta \sqrt{1-k^2 sin^2 w} dw. \quad [26]$$

The eigen strain can be written as (4, 10)

$$\boldsymbol{\epsilon}_1^* = \boldsymbol{S}^{-1}[\boldsymbol{\epsilon} - \boldsymbol{\epsilon}^{\mathbf{0}}], \quad [27]$$

where the background and inclusion strain can be expressed as

$$\boldsymbol{\epsilon^0} = \begin{bmatrix} \epsilon_{11}^0 \\ \epsilon_{22}^0 \\ \epsilon_{33}^0 \\ 0 \\ 0 \\ 0 \end{bmatrix} \quad [28]$$

and

$$\boldsymbol{\epsilon} = \begin{bmatrix} \epsilon_{11} \\ \epsilon_{22} \\ \epsilon_{33} \\ 0 \\ 0 \\ 0 \end{bmatrix}. \quad [29]$$

Another expression of the eigen strain can be written as (11, 12)

$$\boldsymbol{\epsilon_2^*} = \boldsymbol{(S + A)^{-1}} : (-\boldsymbol{\epsilon^0}), \quad [30]$$

where $\boldsymbol{A}$ has been defined in eq. (10).

11. Expression of Eshelby's tensor S for cylindrical inclusion

For cylindrical inclusion with elliptic face of semi axis length a (along x-direction) and semi axis length c (along z-direction), the components of Eshelby's tensor can be written as (9)

$$\boldsymbol{S}_{1111} = \frac{1}{2(1-\nu_b)}\left[\frac{c^2+2ac}{(a+c)^2} + (1-2\nu_b)\frac{c}{a+c}\right], \quad [31]$$

$$\boldsymbol{S}_{2222} = \frac{1}{2(1-\nu_b)}\left[\frac{a^2+2ac}{(a+c)^2} + (1-2\nu_b)\frac{a}{a+c}\right], \quad [32]$$

$$\boldsymbol{S}_{3333} = 0, \quad [33]$$

$$\boldsymbol{S}_{1122} = \frac{1}{2(1-\nu_b)}\left[\frac{c^2}{(a+c)^2} - (1-2\nu_b)\frac{c}{a+c}\right], \quad [34]$$

$$\boldsymbol{S}_{2233} = \frac{1}{2(1-\nu_b)}\frac{2\nu_b a}{a+c}, \quad [35]$$

$$\boldsymbol{S}_{2211} = \frac{1}{2(1-\nu_b)}\left[\frac{a^2}{(a+c)^2} - (1-2\nu_b)\frac{a}{a+c}\right], \quad [36]$$

$$\boldsymbol{S}_{3311} = 0, \boldsymbol{S}_{3322} = 0, \quad [37]$$

$$\boldsymbol{S}_{1212} = \frac{1}{2(1-\nu_b)}\left[\frac{a^2+c^2}{(a+c)^2} + \frac{(1-2\nu_b)}{2}\right], \quad [38]$$

$$\boldsymbol{S}_{1133} = \frac{1}{2(1-\nu_b)}\frac{2\nu_b c}{a+c}, \quad [39]$$

$$\boldsymbol{S}_{2323} = \frac{a}{2(a+c)}, \quad [40]$$

$$\boldsymbol{S}_{3131} = \frac{c}{2(a+c)}. \quad [41]$$

Eshelby's tensor for spherical faced cylindrical inclusion can be found by setting $a = c$.

12. Expression of Eshelby's tensor S for the flat ellipsoid-shaped inclusion

In case of flat shaped inclusion with elliptic face, assuming $a > b >> c$, the I-terms of eqs. 13-16 can be calculated as (9)

$$I_1 = 4\pi\big[\chi(k) - \zeta(k)\big]\frac{bc}{(a^2-b^2)}, \quad [42]$$

$$I_2 = 4\pi\Big[\frac{c}{b}\zeta(k) - \big(\chi(k)-\zeta(k)\big)\frac{bc}{(a^2-b^2)}\Big], \quad [43]$$

$$I_3 = 4\pi\Big[1 - \frac{c}{b}\zeta(k)\Big], \quad [44]$$

$$I_{12} = 4\pi\Big[\frac{c}{b}\zeta(k) - 2\big(\chi(k)-\zeta(k)\big)\frac{bc}{(a^2-b^2)}\Big]/(a^2-b^2), \quad [45]$$

$$I_{23} = 4\pi\Big[1 - 2\frac{c}{b}\zeta(k) + \big(\chi(k)-\zeta(k)\big)\frac{bc}{(a^2-b^2)}\Big]/b^2, \quad [46]$$

$$I_{31} = 4\pi\Big[1 - \frac{c}{b}\zeta(k) - \big(\chi(k)-\zeta(k)\big)\frac{bc}{(a^2-b^2)}\Big]/a^2, \quad [47]$$

$$I_{33} = \frac{4\pi}{3c^2}, \quad [48]$$

where the elliptic integrals χ and ζ are defined as

$$\chi(k) = \int_0^{\frac{\pi}{2}} \frac{dw}{\sqrt{1-k^2 sin^2 w}} \quad [49]$$

$$\zeta(k) = \int_0^{\frac{\pi}{2}} \sqrt{1-k^2 sin^2 w}dw. \quad [50]$$

Using the expressions of the I-terms determined above in eqs. 13-16, the Eshelby's tensor for the flat ellipsoid-shaped inclusion can be determined.

13. Expression of Eshelby's tensor S for the penny-shaped inclusion

For penny-shaped inclusion with radius $a = c >> b$, the components of Eshelby's tensor can be written as (13, p. 81)

$$\boldsymbol{S}_{1111} = \boldsymbol{S}_{2222} = \frac{13-8\nu_b}{32(1-\nu_b)}\pi\frac{b}{a}, \quad [51]$$

$$\boldsymbol{S}_{3333} = 1 - \frac{1-2\nu_b}{1-\nu_b}\frac{\pi}{4}\frac{b}{a}, \quad [52]$$

$$\boldsymbol{S}_{1122} = \boldsymbol{S}_{2211} = \frac{8\nu_b-1}{32(1-\nu_b)}\pi\frac{b}{a}, \quad [53]$$

$$\boldsymbol{S}_{1133} = \boldsymbol{S}_{2233} = \frac{2\nu_b-1}{8(1-\nu_b)}\pi\frac{b}{a}, \quad [54]$$

$$\boldsymbol{S}_{3311} = \boldsymbol{S}_{3322} = \frac{\nu_b}{(1-\nu_b)}\left(1 - \frac{4\nu_b+1}{8\nu_b}\frac{b}{a}\right), \quad [55]$$

$$\boldsymbol{S}_{1212} = \frac{7-8\nu_b}{32(1-\nu_b)}\pi\frac{b}{a}, \quad [56]$$

$$\boldsymbol{S}_{1313} = \boldsymbol{S}_{2323} = \frac{1}{2}\left(1 + \frac{\nu_b-2}{1-\nu_b}\frac{\pi}{4}\frac{b}{a}\right). \quad [57]$$

14. Expressions of ϵ_1^* and ϵ_2^* for spherical inclusion

For spherical inclusion (tumor), the Eshelby tensor's components can be written as (11, 12)

$$S_{1111} = S_{2222} = S_{3333} = m_1 = \frac{7-5\nu_b}{15(1-\nu_b)}, \tag{58}$$

$$S_{1122} = S_{2233} = S_{3311} = S_{2211} = S_{3322} = S_{1133} = m_2 = \frac{5\nu_b - 1}{15(1-\nu_b)}, \tag{59}$$

$$S_{1212} = S_{2323} = S_{3131} = m_3 = \frac{4-5\nu_b}{15(1-\nu_b)}. \tag{60}$$

The eigen strain can be written as (4, 10)

$$\boldsymbol{\epsilon_1^*} = \boldsymbol{S}^{-1}[\boldsymbol{\epsilon} - \boldsymbol{\epsilon^0}]. \tag{61}$$

Using $\epsilon_{11} = \epsilon_{33}, \epsilon_{11}^0 = \epsilon_{33}^0$ for spherical inclusion, the first expression of $\boldsymbol{\epsilon_1^*}$ can be written as

$$\boldsymbol{\epsilon_1^*} = \begin{bmatrix} \frac{m_1(\epsilon_{11}-\epsilon_{11}^0)}{m_1^2+m_1m_2-2m_2^2} - \frac{m_2(\epsilon_{22}-\epsilon_{22}^0)}{m_1^2+m_1m_2-2m_2^2} \\ \frac{(m_1+m_2)(\epsilon_{22}-\epsilon_{22}^0)}{m_1^2+m_1m_2-2m_2^2} - \frac{2m_2(\epsilon_{11}-\epsilon_{11}^0)}{m_1^2+m_1m_2-2m_2^2} \\ \frac{m_1(\epsilon_{11}-\epsilon_{11}^0)}{m_1^2+m_1m_2-2m_2^2} - \frac{m_2(\epsilon_{22}-\epsilon_{22}^0)}{m_1^2+m_1m_2-2m_2^2} \\ 0 \\ 0 \\ 0 \end{bmatrix}. \tag{62}$$

Another expression of the eigen strain can be written as (11, 12)

$$\boldsymbol{\epsilon_2^*} = \boldsymbol{(S+A)^{-1}} : (-\boldsymbol{\epsilon^0}). \tag{63}$$

Let us now expand this equation in terms of the YM and PR of the inclusion and background.

From eq. 63, $\boldsymbol{H} = \boldsymbol{(S+A)^{-1}}$ can be written as

$$\boldsymbol{H} = \begin{bmatrix} D_1 & D_2 & D_2 & 0 & 0 & 0 \\ D_2 & D_1 & D_2 & 0 & 0 & 0 \\ D_2 & D_2 & D_1 & 0 & 0 & 0 \\ 0 & 0 & 0 & 1/(a_3+m_3) & 0 & 0 \\ 0 & 0 & 0 & 0 & 1/(a_3+m_3) & 0 \\ 0 & 0 & 0 & 0 & 0 & 1/(a_3+m_3) \end{bmatrix}, \tag{64}$$

where

$$D_1 = (a_1+a_2+m_1+m_2)/(a_1^2+a_1a_2+2a_1m_1+a_1m_2-2a_2^2+a_2m_1-4a_2m_2+m_1^2+m_1m_2-2m_2^2), \tag{65}$$

$$D_2 = -(a_2+m_2)/(a_1^2+a_1a_2+2a_1m_1+a_1m_2-2a_2^2+a_2m_1-4a_2m_2+m_1^2+m_1m_2-2m_2^2). \tag{66}$$

Here

$$a_1 = \Psi(\lambda_b+2\mu_b)+2\lambda_b\Omega, \tag{67}$$

$$a_2 = \Omega(\Lambda_b+2\mu_b)+\lambda_b\Omega+\lambda_b\Psi, \tag{68}$$

$$a_3 = \mu_b\Phi \tag{69}$$

and

$$\Psi = -(\lambda_b-\lambda_i+\mu_b-\mu_i)/(2\mu_b^2+2\mu_i^2+3\lambda_b\mu_b-3\lambda_b\mu_i-3\lambda_i\mu_b+3\lambda_i\mu_i-4\mu_b\mu_i), \tag{70}$$

$$\Omega = (\lambda_b-\lambda_i)/(2(2\mu_b^2+2\mu_i^2+3\lambda_b\mu_b-3\lambda_b\mu_i-3\lambda_i\mu_b+3\lambda_i\mu_i-4\mu_b\mu_i)), \tag{71}$$

$$\Phi = -\frac{1}{\mu_b-\mu_i}. \tag{72}$$

The expression of $\boldsymbol{\epsilon_2^*}$ can now be obtained as

$$\boldsymbol{\epsilon_2^*} = \begin{bmatrix} -\frac{\epsilon_{11}^0(a_1+m_1)}{L} + \frac{\epsilon_{22}^0(a_2+m_2)}{L} \\ \frac{2\epsilon_{11}^0(a_2+m_2)}{L} - \frac{\epsilon_{22}^0(a_1+a_2+m_1+m_2)}{L} \\ -\frac{\epsilon_{11}^0(a_1+m_1)}{L} + \frac{\epsilon_{22}^0(a_2+m_2)}{L} \\ 0 \\ 0 \\ 0 \end{bmatrix}, \tag{73}$$

where

$$L = a_1^2+a_1a_2+2a_1m_1+a_1m_2-2a_2^2+a_2m_1-4a_2m_2+m_1^2+m_1m_2-2m_2^2. \tag{74}$$

15. Readings from the force sensor in experiments in vivo

Readings from the force sensor in an in vivo elastography experiment is shown in Fig. S18.

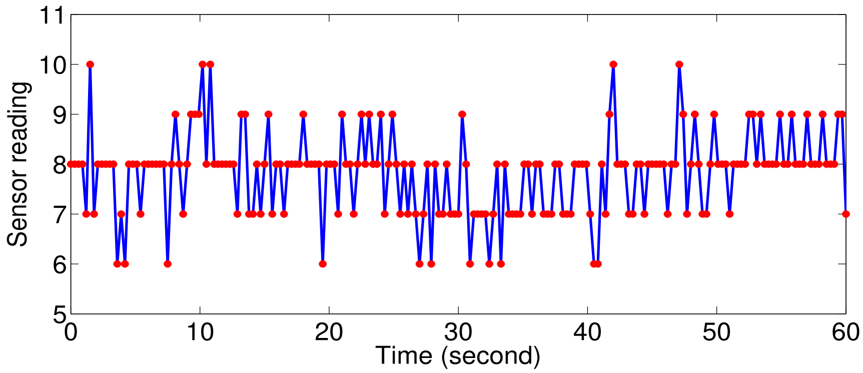

Fig. S18. Reading from the force sensor in an in vivo elastography experiment.

16. Approximation of different shapes with ellipses

In the proposed approach of YM and PR reconstruction, the complex shapes such as tetragon, pentagon and hexagon are approximated with ellipses. These shapes along with the approximated ellipses are shown in Fig. S19.

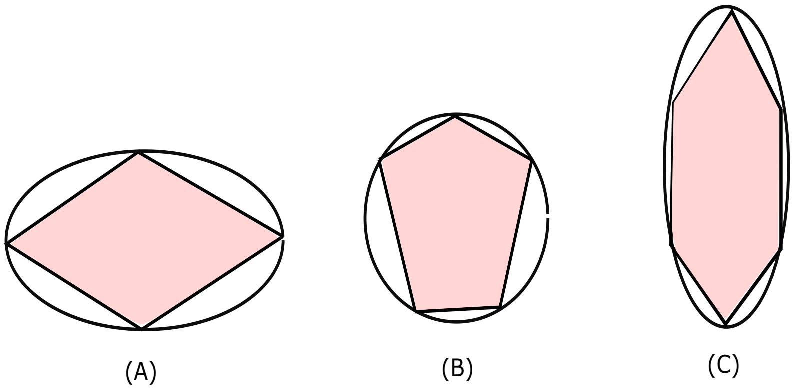

Fig. S19. Approximation of different shapes with ellipses (A) tetragon (B) pentagon (C) hexagon. It has been assumed that in the imaging plane the tumors are of these shapes and if the plane is revolved around the center line, the shape remains the same in all other planes (axisymmetry).

17. Three dimensional samples

A. Cubic samples with elliptical inclusion. We choose three cubic samples (V1-V3) with elliptical inclusion. The three dimensional cubic samples along with the solution space used in the FEA are shown in Fig. S20. The size of the solution space is 2 cm along all three directions. The mesh element has been used in all three dimensional samples is C3D8P. The number of mesh element in the solution space of V1-V3 is 133067. In all the samples, the YM and PR of the normal tissue has been taken as 32.78 kPa and 0.4. The YM and PR of the inclusion has been taken as 97.02 kPa and 0.4. The size of all three dimensional cubic samples is assumed $4 \times 4 \times 4$ cm^3. The lengths of the semi-axes of the inclusions in each sample has been shown in Table S3. The RMSEs in reconstructed YM and PR by the proposed technique with and without axisymmetric assumption in samples V1-V3 are shown in Table S4.

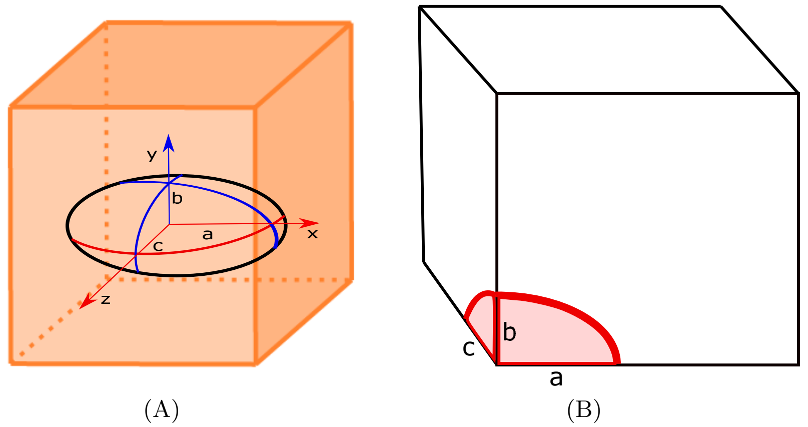

Fig. S20. (A) A cubic sample of a poroelastic material with an elliptical poroelastic inclusion of semi-axes a, b and c along x, y and z direction. The compression is along the negative y-direction. (B) Solution space.

Table S3. Size of the inclusions in samples V1-V3 used in three-dimensional FE simulations

Sample name	a (cm)	b (cm)	c (cm)
V1	0.5	0.25	0.35
V2	0.3	0.9	0.3
V3	0.25	0.35	0.45

Table S4. RMSE (%) in three-dimensional reconstruction and reconstruction by axisymmetric assumption

Sample	3D reconstruction		axisymmetric assumption	
	YM	PR	YM	PR
V1	2.80	4.07	3.10	4.76
V2	3.18	4.01	5.04	4.13
V3	3.51	4.12	5.15	4.23

B. Samples in plane stress and plane strain. Plane strain and plane stress are conditions that may arise in the three-dimensional analysis of the elastic behavior of materials but are of limited relevance for the analysis of tissues. More specifically, plane stress conditions may occur in a sample that is very thin along the z-direction, which is compressed from the x or y direction. In such situation, the stress along the z-direction may be assumed to be zero. On the other hand, plane strain conditions may occur in a cylindrical sample that is infinitely long in the z-direction, and the applied stress is along the periphery of the sample. In such situation, the strain along the z-direction may be assumed to be zero. In elastography applications, plane stress and plane strain conditions are rarely satisfied as most samples have lengths of the same order of magnitude along the three directions. In samples with long cylindrical inclusions such as those considered in Refs. (14–16), the assumption of plane strain inside the inclusion may be justified. However, these cases represent idealized situations that rarely present in experimental applications and have limited relevance in the analysis of the behavior of tumors and biological tissues, in general.

We simulate three-dimensional samples with inclusion in plane stress and plane strain and reconstruct the YM and PR of them by the proposed method. We describe the procedure of simulation and associated results in the following subsections.

B.1. Thin elliptical inclusion (plane stress). We simulate two cubic samples (K1 and K2) with ellipsoidal inclusions of very small thickness along z-direction ($a = 0.3, b = 0.3$ and $c = 0.04$ cm). The number of mesh element in solution space of K1-K2 is 216264. In sample K1, the YM and PR of the inclusion has been taken as 97.02 kPa and 0.4, whereas in sample K2, the YM and PR of the inclusion has been considered 163.90 kPa and 0.4. The YM and PR of the normal tissue is considered as 32.78 kPa and 0.4 in both samples. We show the elevational stress for this sample in Fig. S21 for an applied load of 1 kPa. Very small value of elevational stress inside the inclusion proves that the inclusion is indeed in plane stress condition.

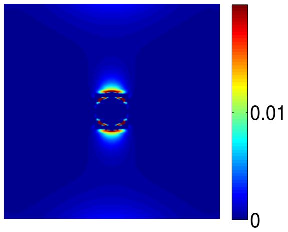

Fig. S21. Elevational stress (kPa) in 3D sample with thin inclusion, which imitates a plane stress condition

Eshleby's tensor For an inclusion with radius $a = b >> c$, which imitates the plane stress condition of the inclusion, the components of Eshelby's tensor can be written as (13, p. 81)

$$\boldsymbol{S}_{1111} = \boldsymbol{S}_{2222} = \frac{13 - 8\nu_b}{32(1-\nu_b)}\pi\frac{c}{a}, \quad [75]$$

$$\boldsymbol{S}_{3333} = 1 - \frac{1-2\nu_b}{1-\nu_b}\frac{\pi}{4}\frac{c}{a}, \quad [76]$$

$$\boldsymbol{S}_{1122} = \boldsymbol{S}_{2211} = \frac{8\nu_b - 1}{32(1-\nu_b)}\pi\frac{c}{a}, \quad [77]$$

$$\boldsymbol{S}_{1133} = \boldsymbol{S}_{2233} = \frac{2\nu_b - 1}{8(1-\nu_b)}\pi\frac{c}{a}, \quad [78]$$

$$\boldsymbol{S}_{3311} = \boldsymbol{S}_{3322} = \frac{\nu_b}{(1-\nu_b)}\left(1 - \frac{4\nu_b + 1}{8\nu_b}\frac{c}{a}\right), \quad [79]$$

$$\boldsymbol{S}_{1212} = \frac{7 - 8\nu_b}{32(1-\nu_b)}\pi\frac{c}{a}, \quad [80]$$

$$\boldsymbol{S}_{1313} = \boldsymbol{S}_{2323} = \frac{1}{2}\left(1 + \frac{\nu_b - 2}{1-\nu_b}\frac{\pi}{4}\frac{c}{a}\right). \quad [81]$$

RMSE in estimated YM and PR RMSE values in estimated YM and PR of K1 and K2 by the proposed technique are tabulated in Table S5.

Table S5. RMSE (%) in estimation of YM and PR of K1-K2

Sample name	YM	PR
K1	5.17	0.18
K2	6.15	1.19

B.2. Infinitely long cylindrical inclusion (plane strain). We simulate two cubic samples (J1 and J2) with infinitely long cylindrical inclusion along z-direction ($a = 0.3, b = 0.3$ cm) as shown in Fig. S22, which imitates the plane strain condition. The number of mesh element in solution space of J1-J2 is 142532. We apply the boundary condition of zero elevational displacement along all the surfaces of the inclusion also to make sure that there is no elevational strain in the inclusion. In sample J1, the YM and PR of the inclusion has been taken as 97.02 kPa and 0.4, whereas in sample J2, the YM and PR of the inclusion has been considered 163.90 kPa and 0.4. The YM and PR of the normal tissue is considered as 32.78 kPa and 0.4 in both samples. We show the elevational strain for this sample in Fig. S23. Very small value of elevational strain inside the inclusion proves that the inclusion is indeed in plane strain condition.

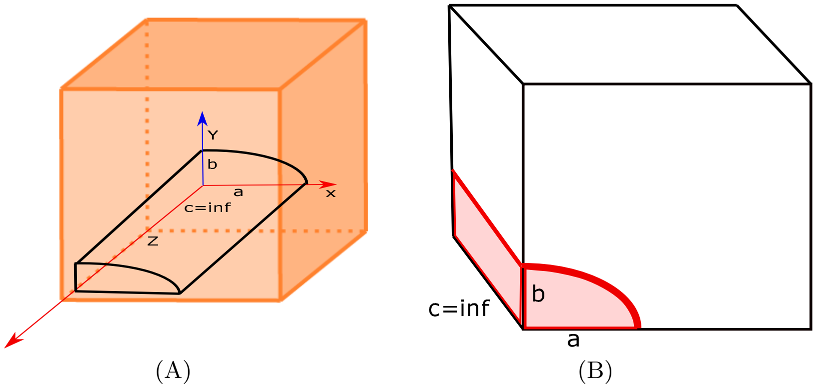

Fig. S22. (A) A schematic of a cubic sample of a poroelastic sample with a cylindrical poroelastic inclusion of elliptical face. (B) Solution space

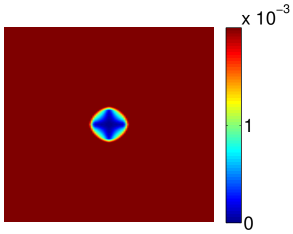

Fig. S23. Elevational strain in a 3D sample with long cylindrical inclusion, which imitates a plane strain condition

Eshleby's tensor For an inclusion with elliptic face of semi-axis length a along x-axis and semi-axis length b along y-axis in plane strain state, the components of Eshelby's tensor can be written as (9)

$$\boldsymbol{S}_{1111} = \frac{1}{2(1-\nu_b)}\left[\frac{b^2+2ab}{(a+b)^2} + (1-2\nu_b)\frac{b}{a+b}\right], \tag{82}$$

$$\boldsymbol{S}_{2222} = \frac{1}{2(1-\nu_b)}\left[\frac{a^2+2ab}{(a+b)^2} + (1-2\nu_b)\frac{a}{a+b}\right], \tag{83}$$

$$\boldsymbol{S}_{3333} = 0, \tag{84}$$

$$\boldsymbol{S}_{1122} = \frac{1}{2(1-\nu_b)}\left[\frac{b^2}{(a+b)^2} - (1-2\nu_b)\frac{b}{a+b}\right], \tag{85}$$

$$\boldsymbol{S}_{2233} = \frac{1}{2(1-\nu_b)}\frac{2\nu_b a}{a+b}, \tag{86}$$

$$\boldsymbol{S}_{2211} = \frac{1}{2(1-\nu_b)}\left[\frac{a^2}{(a+b)^2} - (1-2\nu_b)\frac{a}{a+b}\right], \tag{87}$$

$$\boldsymbol{S}_{3311} = 0, \boldsymbol{S}_{3322} = 0, \tag{88}$$

$$\boldsymbol{S}_{1212} = \frac{1}{2(1-\nu_b)}\left[\frac{a^2+b^2}{(a+b)^2} + \frac{(1-2\nu_b)}{2}\right], \tag{89}$$

$$\boldsymbol{S}_{1133} = \frac{1}{2(1-\nu_b)}\frac{2\nu_b b}{a+b}, \tag{90}$$

$$\boldsymbol{S}_{2323} = \frac{a}{2(a+b)}, \tag{91}$$

$$\boldsymbol{S}_{3131} = \frac{b}{2(a+b)}. \tag{92}$$

RMSE in the estimated YM and PR RMSE values in the estimated YM and PR of J1 and J2 by the proposed technique are tabulated in Table S6.

Table S6. RMSE (%) in the estimation of YM and PR of J1-J2

Sample name	YM	PR
J1	3.67	1.32
J2	5.82	2.18

C. Samples with multiple inclusions. We simulate four cubic samples (U1-U4) with three inclusions inside it as shown in Figs. S24 and S25. The central, right (top) and left (bottom) inclusions are numbered as 1, 2 and 3. All inclusions are assumed spherical. The radius of inclusion 1 is assumed $a_1 = 0.3$ cm and of inclusion 2 and 3 is assumed $a_2 = 0.2$ cm. The distance between inclusion 1 to other two inclusions is $d = 0.65$ cm. The material properties of the inclusions of U1-U4 are tabulated in Table S7. The YM of the background is taken as 32.78 kPa in all the samples. The PR of the background of U1 and U3 is taken as 0.4 and of U2 and U4 is taken as 0.3. The RMSEs in the reconstructed YM and PR by the proposed method in the inclusions in these samples are tabulated in Table S8.

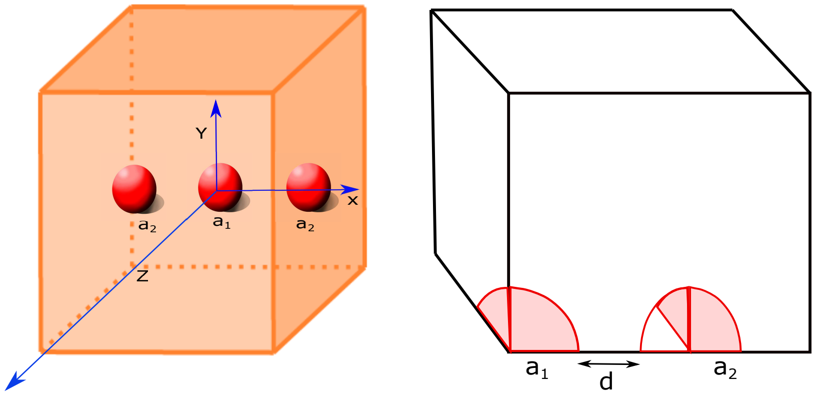

(A) (B)

Fig. S24. (A) A schematic of a cubic sample (U1-U2) of a poroelastic material with multiple spherical poroelastic inclusions side by side. The axial direction is along the y-axis. (B) Solution space

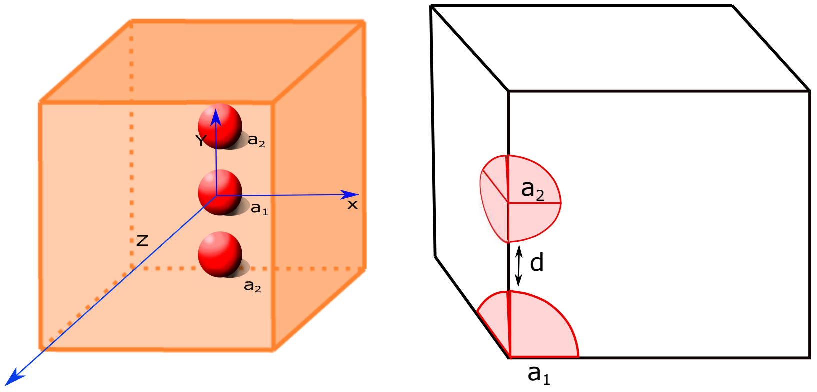

(A) (B)

Fig. S25. (A) A schematic of a cubic sample (U3-U4) of a poroelastic material with multiple spherical poroelastic inclusions on top of each other. The axial direction is along the y-axis. (B) Solution space

Table S7. Material properties of U1-U4

Sample	inclusion 1		inclusion 2		inclusion 3	
	YM	PR	YM	PR	YM	PR
U1	97.02	0.4	65.56	0.4	65.56	0.4
U2	163.90	0.3	81.95	0.3	81.95	0.3
U3	97.02	0.4	65.56	0.4	65.56	0.4
U4	163.90	0.3	81.95	0.3	81.95	0.3

Table S8. RMSE (%) in estimation of YM and PR of U1-U4

Sample	inclusion 1		inclusion 2		inclusion 3	
	YM	PR	YM	PR	YM	PR
U1	0.7	1.07	4.39	3.80	5.49	2.17
U2	0.6	1.33	7.67	8.00	8.54	5.67
U3	10.96	0.51	0.81	0.01	3.33	0.27
U4	10.11	0.72	0.62	0.02	2.83	0.21

18. Experimental setup for controlled elastography experiments

The experimental setup for the controlled elastography experiments is shown in Fig. S26. Different components of the experimental setup are shown in this figure.

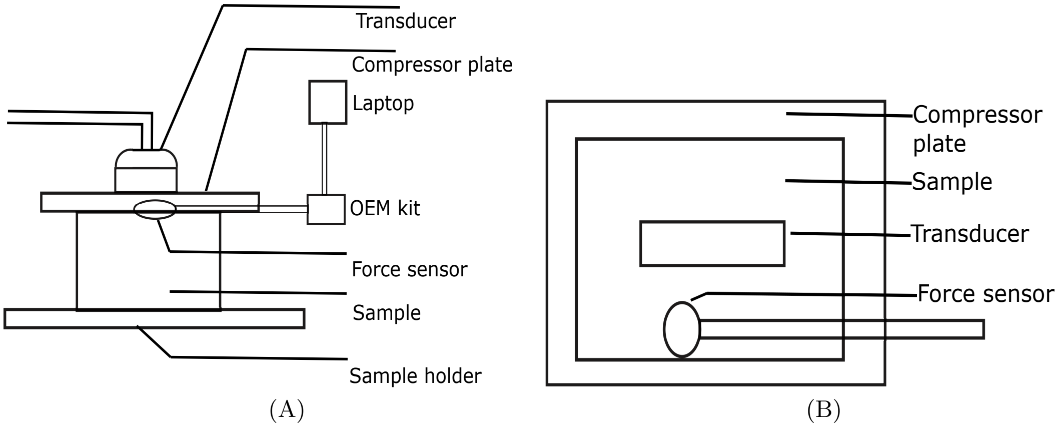

Fig. S26. (A) Experimental setup (B) Top view of the setup showing the placement of the force sensor between the compressor plate and sample

19. Assumption of zero shear strain and stress

For uniaxial stress, we assumed in our reconstruction method that the shear stress is zero both in the normal tissue and inside the tumor. This can be validated by the shear strain found in the untreated tumor cases as shown in Fig. S27. We see from this figure that shear strain is much smaller than the axial and lateral strains in these cases, which proves that the assumption of zero shear stress is valid (shear stress=shear strain $\times$ shear modulus).

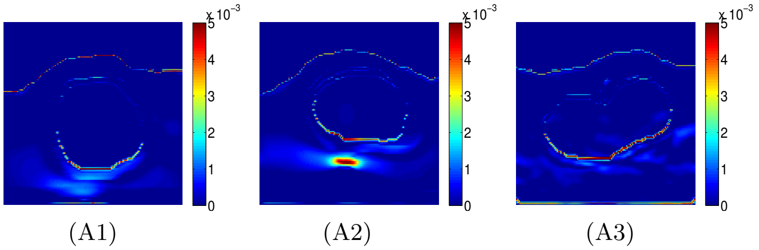

Fig. S27. (A1-A3) axial shear strains in case of 3 untreated tumors in third week.

20. Controlled experiments on breast phantom

We used the breast phantom model 059 from Computerized Imaging Reference Systems (CIRS), Inc., Norfolk, VA, USA. As provided by the manufacturer, in this phantom, the Young's modulus of each inclusion mass is around 50 kPa, while the background has a Young's modulus of 20 ± 5 kPa (17, 18). The PR of both inclusions and background of this phantom is 0.5 (19). The applied compression was evaluated using a graphical user interface monitoring the force sensor. The axial and lateral strain was estimated using the pre- and post-compressed ultrasound radio frequency data acquired in the elastography experiments.

Fig. S28 shows selected results from controlled experiments obtained from a breast-mimicking phantom containing different spherical inclusions simulating tumors with similar stiffness but different size. In Fig. S28, the estimated axial strain, lateral strain, reconstructed YM and PR distributions for one of the inclusions inside the breast phantom are shown.

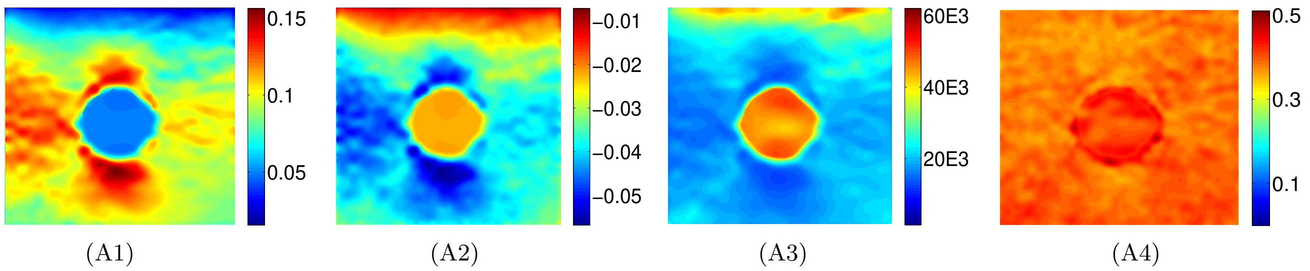

Fig. S28. Estimated axial strain (A1), lateral strain (A2), YM image (A3) and PR image (A4) from the controlled experiment (CE1). This figure shows results for applied compression of 1.88 kPa. The estimated YM is in the range of $45 - 51$ kPa in the inclusion and in the range of $17 - 21$ kPa in the background region. The estimated PR is around 0.45 in the inclusion region and 0.42 in the background region.

21. Calculation of stress

FlexiForce OEM Development Kit manufactured by Tekscan, Inc., South Boston, MA, USA-02127 was employed to inspect and adjust the applied compression in both the controlled and in vivo experiments. A Microsoft Windows based interface software is provided with the sensor and can be used to observe and record the applied force. A temporal curve showing the applied compression in one of the in vivo experiments is reported in Fig. S18. The sensor used in the kit is model #A201, which senses a force range $0 - 4.4$ N in a scale of $0 - 255$. The diameter of the sensing area of the sensor is 9.53 mm. The area of the sensing area is calculated as 7.1331×10^{-5} m^2 ($A_r = \pi r^2$). The applied pressure in Pa is calculated using

$$\sigma_0 = \frac{F_r \times 4.4}{255 \times A_r}, \quad [93]$$

where F_r is the mean force reading obtained from the sensor during the experiments. It should be noted that σ_0 is the axial component of $\boldsymbol{\sigma^0}$ in eq. 3 and other two components (lateral and elevation) of $\boldsymbol{\sigma^0}$ are zero.

22. Computational aspect of the proposed method

The proposed formulation for the estimation of YM and PR of inclusions located inside a background material is well-posed and computationally fast. The method requires solving a single optimization problem. We propose a straightforward formulation, which can be implemented using commercially available computational software (Matlab, Mathematica, C, Java, Python, etc.). The method can also be easily parallelized or converted in tensor format, which would make it suitable for implementation using tensorflow or other modern frameworks. This would increase the computation speed of the proposed method substantially. Below we discuss the computation of different parameters necessary in our formulation.

Estimation of axial and lateral displacements and strains. To compute the elastograms from the pre- and post-compressed RF data in simulations and experiments, we used the method described in (8). The method in (8) is a two-step method, which uses dynamic programming elastography (DPE) and Horn-Schunck optical flow estimation (HS). In our study, to compute the elastograms with the DPE, the range of variation of axial displacement was set to 0 to -60 data points, and the range of variation of lateral displacement was set to -4 to 4 data points. The values of regularization weights along the axial and lateral directions, α_a and α_l were set to 0.15. For estimating the strains by HS, the trade-off parameter β was assumed 1. The number of pyramid levels was assumed 4, and the maximum number of warping per pyramid level was set to 3. To warp and up-scale from coarse to fine scales, a bi-cubic interpolation was used on the pre- and post-compression RF data.

We note that a strain tensor ϵ and $\epsilon_{\mathbf{0}}$ has six components. The diagonal components are the axial, lateral and elevational normal strains and the other components are the shear strains. As in our experiments the applied stress is uniaxial and the lesion is bounded to the background, the shear components of the strain (as well as stress) tensors are assumed to be zero. Therefore, ϵ and $\epsilon_{\mathbf{0}}$ have only three non-zero components. To justify this assumption, we show shear strain images for three untreated tumors with associated discussion (Fig. S27). We see from these images that shear strains are negligible.

Estimation of YM and PR by the proposed method. The axisymmetric assumption in eq. (6) produces insignificant error ($< 5.2\%$) even when lengths of all three semi-axes of the elliptical inclusion are different from each other, which has been proven in section 17-A. Therefore, we used eq. (6) for reconstructing the YM and PR of all our samples from controlled and in vivo experiments. For the same reason, we simulated only axisymmetric samples for the finite element and ultrasound simulation study. For FE and ultrasound simulation data, we select a square region of 5×5 pixels in the left corner of the axial and lateral strain elastograms (128×128 pixels). The mean strains of this area are assumed to be representative of the axial and lateral strains of the background region. For the in vivo and controlled experiments, we choose a square region of 10×10 pixels in the normal tissue/background region and compute the mean values of the strains in that region. If any other organ is present in the imaging region of the sample in vivo, we avoided the region and did not include strains from that region in computing the strains in normal tissue.

We formulate the inverse reconstruction formula using Eshelby's theory for each pixel inside the inclusion, which allows us to compute the YM and PR for samples with heterogeneous elastic properties inside the inclusion. Every pixel inside the inclusion is assumed to be independent, and the YM and PR is computed for each pixel without using any information from other pixels inside the inclusion. The YM and PR of the background are assumed to be same in the reconstruction process of YM and PR for all the pixels in the inclusion.

For estimation of YM and PR using the proposed method, non linear least square optimization by 'trust-region-reflective' algorithm in MATLAB (The MathWorks, Natick, MA) is used to minimize the cost function J in eq. 6, where the maximum number of iteration is set to 100. Complex shapes such as tetragon, pentagon and hexagon are approximated with ellipses, and the cost function for the elliptical tumor has been used for these shapes. The approximation of these complex shapes with ellipses are shown in Fig. S19. The lower and higher limits for the YM are set to $0.1 \times \frac{\sigma_0}{\epsilon_{zz}}$ and $100 \times \frac{\sigma_0}{\epsilon_{zz}}$ inside the tumor. The lower limit of PR in the cost function minimization process is set to $-0.8 \times \frac{\epsilon_{xx}}{\epsilon_{zz}}$ and the higher limit is set to 0.495. Here, ϵ_{zz} and ϵ_{xx} are axial and lateral strains, respectively.

23. Additional YM reconstruction methods used for statistical comparison

We used the formulation provided in Shin et al. (10) and Bilgen et al. (20) to implement the 3DB and 3DS methods, respectively. The formulations of these methods are briefly described in below. For the 3DS method (10), the formulation

for PR values different than 0.45 is not provided. Although using the 3DB method, it is possible to estimate the YM of the inclusion for other PR values (20), we see that the YM value depends non-linearly to the PR. There is no straightforward way to compute the error in the YM estimation, when the actual PR value deviates from the one assumed by the model. The correctness of the implementations of these methods is verified by matching the results with those reported in the corresponding papers for the same simulation conditions.

In the 3DB method (20), the axial strain inside the inclusion can be written as

$$\epsilon_{22,i} = \frac{\sigma_0(1-\nu_b)}{2(1+\nu_b)}\left(\frac{10(1+\nu_b)}{(7-5\nu_b)\mu_b+(8-20\nu_b)\mu_i} + \frac{1-2\nu_i}{(2-4\mu_i)\mu_b+(1+\nu_i)\mu_i}\right), \quad [94]$$

$$\epsilon_{22,b} = \frac{\sigma_0}{2(1+\nu_b)\mu_b}. \quad [95]$$

Using $\nu_b = \nu_i = 0.5$ and taking their ratio, we obtain

$$\frac{\epsilon_{22,b}}{\epsilon_{22,i}} = 0.4\frac{E_i}{E_b} + 0.6 \quad [96]$$

$$\frac{E_i}{E_b} = 2.5\frac{\epsilon_{22,b}}{\epsilon_{22,i}} - 1.5. \quad [97]$$

In the 3DS method (10), for a nearly incompressible inclusion with PR of 0.45, the ratio of E_i and E_b can be written as

$$\begin{aligned}\frac{E_i}{E_b} &= p_{00} + p_{10}x + p_{01}y + p_{20}x^2 + p_{11}xy + p_{02}y^2 \\ &= +p_{30}x^3 + p_{21}x^2y + p_{12}xy^2 + p_{40}x^4 + p_{31}x^3y + p_{22}x^2y^2,\end{aligned} \quad [98]$$

where $x = \frac{a}{c}, y = \frac{\epsilon_{22,b}}{\epsilon_{22,i}}$ and $p_{00} = -2.439, p_{10} = -0.4783, p_{01} = 4.827, p_{20} = 0.747, p_{11} = -1.887, p_{02} = -0.2935, p_{30} = -0.1538, p_{21} = 0.3153, p_{12} = 0.166, p_{40} = 0.00832, p_{31} = -0.0136, p_{22} = -0.0135$. Here, a and c are the lengths of major and minor axes of the elliptic inclusion.

24. Calculation of RMSE of estimated YM and PR

RMSE was computed on a pixel by pixel basis. The squared errors were computed by calculating the square of the difference between the actual values of YM and PR and their reconstructed YM and PR values for each pixel inside the tumor. Then mean value of all the squared errors was computed. The RMSE was obtained by taking the square root of the mean error value.

Calculation of RMSE for the estimated YM and PR of the inclusion was performed using the following formula (8).

$$\mathrm{RMSE} = \sqrt{\frac{\sum_n^N (\Lambda_e(n) - \Lambda_t(n))^2}{N}} \times \frac{100 \times N}{\sum_n^N \Lambda_t(n)}, \quad [99]$$

where Λ_e is the vectorized (reshaped from 2D to 1D) YM or PR of the inclusion from YM and PR images estimated by different methods and Λ_t is the vectorized true YM or PR of the inclusion. N is the total number of points inside the inclusion of the estimated YM or PR image.

25. Specifications of the samples

The material properties of all samples are chosen based on values of YM and PR for tumors and normal tissues available in the literature (2, 21–24). In samples A-M, the normal tissues have YM equal to 32.78 kPa. A, D, E and F have tumors with YM equal to 97.02 kPa. B, C, H, I, J, K, L and M have tumors with YM equal to 50, 163.90, 491.7, 819.5, 1639, 3278, 16.39, 6.556 and 3.78 kPa, respectively. The PR of normal tissues in A, B and C is equal to 0.49, in D is equal to 0.45 and in E-M is equal to 0.2. The PR of tumor is equal to 0.4 in A-C, 0.45 in D and E, 0.3 in F, 0.45 in G-J and 0.3 in K-M. These mechanical parameters are shown in tabulated form in Table S1.

For samples Z1-Z8, H1-H3, B1-B3 and R1-R4, the YM and PR of tumor are set to 97.02 kPa and 0.3, whereas the YM and PR of the normal tissue are set to 32.78 kPa and 0.2. For samples X1-X9, the YM and PR of tumor are set to 97.02 kPa and 0.45 and the YM and PR of the normal tissue are set to 32.78 kPa and 0.2.

All the samples simulated are of 4 cm height and 2 cm width in an axisymmetric setup. In samples A-M and X1-X9, the radius of the spherical inclusion is 0.3 cm. In sample Z1, the radius of the inclusion is 0.3 cm, the lengths of elliptical axes along lateral and axial direction in inclusions of samples Z2 and Z3 are 0.2 cm and 0.5 cm and 0.5 cm and 0.2 cm, respectively. The radius and height of the cylindrical inclusion of sample Z4 are 0.3 cm and 0.55 cm. The radius of the penny-shaped inclusion of sample Z5 is 0.5 cm and the height is 0.05 cm. The length of each side of tetragonal, pentagonal and hexagonal inclusions in samples Z6, Z7 and Z8 are 0.45 cm. The dimensions of the inclusions and boundary conditions of samples B1-B3, H1-H3 and R1-R4 are described in detail in supplementary material.

26. Mechanical measurements

Mechanical measurements of the YM of drained cylindrical tofu and polyacrylamide samples were performed using a simple performance tester (Controls Group USA, Inc., Elgin, IL). Polyacrylamide gel was prepared using Acrylamide (40% v/v), bisacrylamide (4% v/v), ammonium persulfate and tetramethylethylenediamine following the method described in (25). Six cylinders of two different types of tofus (diameter ≈3 inch, height 6 inch) and polyacrylamide (diameter ≈4 inch, height 6 inch) were placed on an impermeable plate and tested in unconfined configuration using flow time testing configuration for 2 minutes. The maximum axial strain was set in the range 1% - 10%. The acquired steady state strain versus stress curves were then used to estimate the YM as detailed in (26). To determine the PR of the samples, a small compression was applied on the samples using the same compression apparatus used for imaging. The corresponding changes in the axial and lateral length of the samples were estimated using a digital Vernier caliper. The PR of the samples was estimated as the ratio of the lateral and axial strains, due to the applied compression.

27. Surface area and solidity of tumors

The surface area of the tumor, A_s is calculated in cm^2 as

$$A_s = \frac{n_p \times 15.2}{n_t}, \qquad [100]$$

where n_p is the pixel number inside cancer tumor and n_t is the total number of pixels in the axial strain elastograms. The total area of the elastograms was calculated 15.2 cm^2 using length of 4 cm and width of 3.8 cm.

The solidity of the tumor is calculated as (27)

$$s_t = \frac{a}{c_a}, \qquad [101]$$

where a is the area and c_a is the convex area of the tumor.

Mean surface areas of the tumors with the corresponding standard deviations for all treated and untreated mice at three time points are shown in Fig. S29 (A1). The mean surface area of the treated tumors does not change significantly with time, whereas the mean surface area of the untreated tumors increases with time.

The solidity of the tumor is a measure of the regularity of the shape of the tumor, and mean value of the solidity at the three time points for all tumors is shown in Fig. S29 (A2) . Solidity is higher in the case of the treated tumors than in the case of the untreated tumors at all time points. In previous studies, low values of solidity have been associated to malignancy (28).

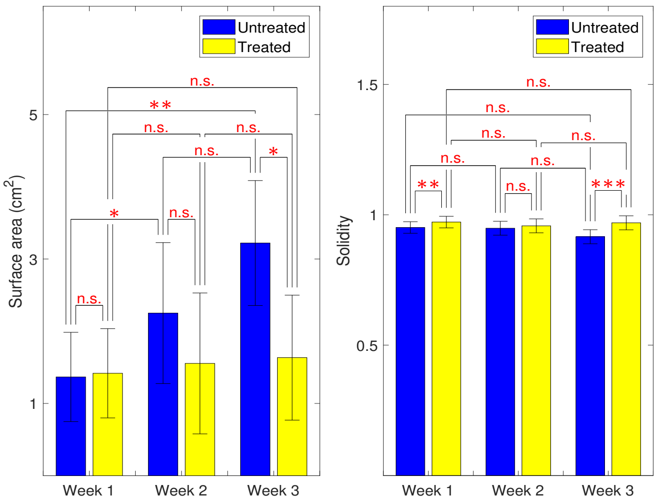

(A1) (A2)

Fig. S29. (A1) Mean surface areas of the tumors for the treated and untreated mice at week 1, week 2 and week 3. (A2) Mean values of solidity for all treated and untreated mice at week 1, week 2 and week 3. n.s. means not statistically significant. One, two and three stars corresponds to p-value less than $0.05, 0.01, 0.001$, respectively. The mean value of surface area of the tumors increases from week 1 to week 3 for untreated mice and remains almost constant for the treated ones. Mean value of solidity of the tumors is consistently higher for the treated tumors than the untreated ones.

28. Data acquisition parameters and image resolution

In our experiments, we used a linear array transducer with 6.6 MHz center frequency, 50% bandwidth, 1mm beamwidth at the focus. This acquisition configuration is commonly used for imaging lesions/tumors using ultrasound elastography in both proof of principle and clinical studies as shown by different labs (29–32). This acquisition configuration allows generation of elastograms with a theoretical spatial resolution in the range 0.3-0.5 mm (33). However, our working resolution is worse than the theoretical one and depends on the strain estimation method (34, 35). In the case of the strain estimation method used in this study, which is based on sample tracking, the working resolution of the strain images is estimated to be about 1mm (8). This resolution was found to be acceptable for the tumors imaged in this study (the size of the tumors ranged from 0.5cm to 2cm). The reconstructed YM and PR images from the tumors appear smoother than the strain images due to the use of spatial filters needed to avoid errors in the reconstructed parameters (8). We note that, qualitatively, the spatial resolution of our reconstructed YM and PR images is comparable to the spatial resolution of YM images reconstructed with previously proposed methods (15, 36). The use of ultrasonic systems with higher bandwidths and smaller beamwidths improves the quality of axial and lateral strain estimates (34, 37) and, therefore is expected to improve the quality of the YM and PR reconstructed images as well.

1. Islam MT, Chaudhry A, Unnikrishnan G, Reddy J, Righetti R (2018) An analytical model of tumors with higher permeability than surrounding tissues for ultrasound elastography imaging. *Journal of Engineering and Science in Medical Diagnostics and Therapy* 1(3):031006031006.
2. Leiderman R, Barbone PE, Oberai AA, Bamber JC (2006) Coupling between elastic strain and interstitial fluid flow: Ramifications for poroelastic imaging. *Physics in medicine and biology* 51(24):6291.
3. (2019) Detect Cell Using Edge Detection and Morphology - MATLAB & Simulink Example (https://www.mathworks.com/help/images/detecting-a-cell-using-image-segmentation.html).
4. Eshelby JD (1957) The determination of the elastic field of an ellipsoidal inclusion, and related problems in *Proceedings of the Royal Society of London A: Mathematical, Physical and Engineering Sciences*. (The Royal Society), Vol. 241, pp. 376–396.
5. Desai RR, Krouskop TA, Righetti R (2010) Elastography Using Harmonic Ultrasonic Imaging: A Feasibility Study. *Ultrasonic Imaging* 32(2):103–117.
6. Chaudhry A, Unnikrishnan G, Reddy J, Krouskop TA, Righetti R (2013) Effect of permeability on the performance of elastographic imaging techniques. *IEEE transactions on medical imaging* 32(2):189–199.
7. Righetti R, Ophir J, Srinivasan S, Krouskop TA (2004) The feasibility of using elastography for imaging the Poisson's ratio in porous media. *Ultrasound in medicine & biology* 30(2):215–228.
8. Islam MT, Chaudhry A, Tang S, Tasciotti E, Righetti R (2018) A new method for estimating the effective Poisson's ratio in ultrasound poroelastography. *IEEE transactions on medical imaging* 37(5):1178–1191.
9. Weinberger C, Cai W (2004) *Lecture Note 2. Eshelby's Inclusion I.* (Stanford University).
10. Shin B, Gopaul D, Fienberg S, Kwon HJ (2016) Application of Eshelby's Solution to Elastography for Diagnosis of Breast Cancer. *Ultrasonic imaging* 38(2):115–136.
11. Ju J, Sun L (2001) Effective elastoplastic behavior of metal matrix composites containing randomly located aligned spheroidal inhomogeneities. Part I: Micromechanics-based formulation. *International Journal of Solids and Structures* 38(2):183–201.
12. Ju J, Sun L (1999) A novel formulation for the exterior-point Eshelby's tensor of an ellipsoidal inclusion. *Journal of Applied Mechanics* 66(2):570–574.
13. Mura T (1987) *Micromechanics of Defects in Solids. Mechanics of Elastic and Inelastic Solids, Second, Revised Edition.* (Martinus Nijhoff Publishers, Dordrecht, The Netherlands).
14. Richards MS, Barbone PE, Oberai AA (2009) Quantitative three-dimensional elasticity imaging from quasi-static deformation: A phantom study. *Physics in Medicine & Biology* 54(3):757.
15. Doyley M, Meaney P, Bamber J (2000) Evaluation of an iterative reconstruction method for quantitative elastography. *Physics in medicine and biology* 45(6):1521.
16. Kallel F, Bertrand M, Ophir J (1996) Fundamental limitations on the contrast-transfer efficiency in elastography: An analytic study. *Ultrasound in Medicine & Biology* 22(4):463–470.
17. Yue J, et al. (2015) Comparison between 3D Supersonic Shear Wave Elastography and Magnetic Resonance Elastography: A preliminary experimental study in *Journées RITS 2015*. pp. pp–142.
18. Cournane S, Fagan A, Browne J (2012) Review of ultrasound elastography quality control and training test phantoms. *Ultrasound* 20(1):16–23.
19. Hollerieth K, et al. (2016) Preclinical evaluation of acoustic radiation force impulse measurements in regions of heterogeneous elasticity. *Ultrasonography* 35(4):345.
20. Bilgen M, Insana MF (1998) Elastostatics of a spherical inclusion in homogeneous biological media. *Physics in Medicine and Biology* 43(1):1.
21. Stylianopoulos T, et al. (2013) Coevolution of solid stress and interstitial fluid pressure in tumors during progression: Implications for vascular collapse. *Cancer research* 73(13):3833–3841.
22. Mpekris F, Baish JW, Stylianopoulos T, Jain RK (2017) Role of vascular normalization in benefit from metronomic chemotherapy. *Proceedings of the National Academy of Sciences* 114(8):1994–1999.
23. Fung YC (1993) Mechanical properties and active remodeling of blood vessels in *Biomechanics*. (Springer), pp. 321–391.
24. Netti PA, Baxter LT, Boucher Y, Skalak R, Jain RK (1997) Macro-and microscopic fluid transport in living tissues: Application to solid tumors. *AIChE journal* 43(3):818–834.
25. Chaudhry A, Yazdi IK, Kongari R, Tasciotti E, Righetti R (2016) A new class of phantom materials for poroelastography imaging techniques. *Ultrasound in medicine & biology* 42(5):1230–1238.
26. Krouskop TA, Wheeler TM, Kallel F, Garra BS, Hall T (1998) Elastic moduli of breast and prostate tissues under compression. *Ultrasonic imaging* 20(4):260–274.
27. (2019) Measure properties of image regions - MATLAB regionprops (https://www.mathworks.com/help/images/ref/regionprops.html).
28. Tahmasbi A, Saki F, Shokouhi SB (2011) Classification of benign and malignant masses based on Zernike moments. *Computers in biology and medicine* 41(8):726–735.
29. Hiltawsky KM, et al. (2001) Freehand ultrasound elastography of breast lesions: Clinical results. *Ultrasound in Medicine & Biology* 27(11):1461–1469.
30. Tan SM, Teh HS, Mancer JFK, Poh WT (2008) Improving B mode ultrasound evaluation of breast lesions with real-time ultrasound elastography—A clinical approach. *The Breast* 17(3):252–257.
31. Zhi H, et al. (2007) Comparison of ultrasound elastography, mammography, and sonography in the diagnosis of solid breast lesions. *Journal of ultrasound in medicine* 26(6):807–815.
32. Gong X, Wang Y, Xu P (2013) Application of Real-time Ultrasound Elastography for Differential Diagnosis of Breast Tumors. *Journal of Ultrasound in Medicine* 32(12):2171–2176.
33. Righetti R, Ophir J, Ktonas P (2002) Axial resolution in elastography. *Ultrasound in Medicine & Biology* 28(1):101–113.
34. Srinivasan S, Righetti R, Ophir J (2003) Trade-offs between the axial resolution and the signal-to-noise ratio in elastography. *Ultrasound in medicine & biology* 29(6):847–866.
35. Srinivasan S, Righetti R, Ophir J (2004) An experimental characterization of elastographic spatial resolution: Analysis of the trade-offs between spatial resolution and contrast-to-noise ratio. *Ultrasound in Medicine & Biology* 30(10):1269–1280.
36. Sebag F, et al. (2010) Shear wave elastography: A new ultrasound imaging mode for the differential diagnosis of benign and malignant thyroid nodules. *The Journal of Clinical Endocrinology & Metabolism* 95(12):5281–5288.
37. Righetti R, Srinivasan S, Ophir J (2003) Lateral resolution in elastography. *Ultrasound in Medicine & Biology* 29(5):695–704.
